# Mechanistic basis for relaxation of DNA supercoils by human topoisomerase IIIα–RMI1–RMI2

**DOI:** 10.1073/pnas.2406949123

**Published:** 2026-01-23

**Authors:** Dian Spakman, Andreas S. Biebricher, Anna H. Bizard, Ian D. Hickson, Erwin J. G. Peterman, Gijs J. L. Wuite, Graeme A. King

**Affiliations:** ^a^Department of Physics and Astronomy, and LaserLaB Amsterdam, Vrije Universiteit Amsterdam, Amsterdam 1081 HV, The Netherlands; ^b^Department of Cellular and Molecular Medicine, Center for Chromosome Stability, University of Copenhagen, Copenhagen N 2200, Denmark; ^c^Institute of Structural and Molecular Biology, Division of Biosciences, University College London, London WC1E 6BT, United Kingdom

**Keywords:** topoisomerase, supercoiling, optical tweezers, fluorescence imaging

## Abstract

Topoisomerase enzymes are essential for the regulation of DNA topology. Human topoisomerase IIIα is a Type 1A topoisomerase that exists as a complex with RMI1 and RMI2, known as TRR. The TRR complex can unlink entwined DNA strands and is known to be important for resolving DNA replication and recombination intermediates. It has recently been proposed that TRR can also relax transient negatively supercoiled loops of DNA generated by the translocase PICH and that this activity may help to facilitate the resolution of ultrafine anaphase bridges (UFBs) between segregating sister chromatids. However, the mechanism by which TRR interacts with, and processes, negatively supercoiled DNA is not well understood. Here, we establish a single-molecule strategy to simultaneously measure real-time changes in supercoiling density and visualize the interactions of TRR with underwound DNA using a combination of optical tweezers and fluorescence imaging. We demonstrate that TRR relaxes highly negatively supercoiled DNA in a processive manner and that the timescale for relaxation is less than the expected lifetime of the negatively supercoiled loops generated by PICH. We also show that in the absence of free protein in solution, TRR remains bound to the DNA for long time periods after the torsional stress has been released. Our findings provide a mechanistic basis for how TRR can relax negative supercoils, consistent with its proposed role in UFB resolution. Moreover, our assay could also be widely applied to study the interactions of other families of topoisomerases with negatively supercoiled DNA.

## Significance

The human topoisomerase IIIα–RMI1–RMI2 complex (TRR) is important for resolving entangled DNA structures known as ultrafine anaphase bridges (UFBs) during mitosis. Recent evidence suggests that this may involve the relaxation of negative supercoils generated by the protein PICH. Here, we report a single-molecule assay to measure supercoil relaxation in real time while imaging the binding of topoisomerases. Using this approach, we demonstrate that TRR can relax negative supercoils faster than they are expected to be generated by PICH. This deepens our mechanistic understanding of how TRR may facilitate UFB resolution. Moreover, we propose that our assay could be implemented to correlate protein dynamics with supercoil relaxation for other topoisomerase enzymes.

Topoisomerase enzymes are essential for regulating the topological state of DNA during processes such as DNA replication, transcription, and recombination (1–3). They achieve this by creating a transient break in DNA, through which another segment of DNA can pass. Topoisomerases can be classified into two families, referred to as Type 1 and Type 2, depending on whether they cleave one strand or both strands of the DNA (4). Both families can be further divided into different subfamilies. Type 1 topoisomerases consist of three subfamilies, known as Type 1A, Type 1B, and Type 1C (4, 5). Type 1A subfamily members are found in all domains of life and often play key roles in either maintaining supercoiling homeostasis or untangling (i.e., decatenating) entwined DNA structures (6–9). These enzymes require the presence of single-stranded (ss)DNA for catalytic activity (10–12), which often arises in negatively supercoiled (i.e., underwound) DNA (13–17) as well as in several replication/recombination intermediates, such as hemicatenanes (18–22).

Type 1A topoisomerases exhibit a highly conserved toroidal structure and alter the topological state of DNA using an enzyme-bridged strand-passage mechanism (3, 7–9), involving the following steps. First, the enzyme cleaves the ssDNA backbone, creating a covalent bond with the 5′-end of the DNA and a noncovalent interaction with the 3′-end (23–26). This generates an enzyme-bridged gate in the ssDNA that can open and close due to conformational changes in the enzyme (27–32). Upon opening of the gate, a second DNA segment (referred to as the transfer or T-strand) is able to enter the central cavity of the toroidal fold of the enzyme (28, 32). Once the second strand enters the cavity, the gate closes and the ssDNA backbone is religated, resulting in a change in the DNA linking number (*Lk*) of one (33–35).

Apart from reverse gyrases (36), Type 1A topoisomerases are ATP-independent and are often classified into two main subgroups, TopoI and TopoIII (4), named after the Type 1A topoisomerases in *Escherichia coli*. TopoI enzymes are, in general, more efficient at relaxing supercoils than (de)catenating DNA, whereas the opposite is typically the case for TopoIII enzymes (11, 37, 38). While many bacteria possess both TopoI and TopoIII, eukaryotes have only TopoIII (4), which often exists in complex with one or more OB-fold regulatory protein(s) (39–42). Higher eukaryotes typically encode two TopoIII enzymes, known as TopoIIIα and TopoIIIβ (4). TopoIIIα forms a stable complex with the OB-fold proteins RMI1 and RMI2, known as TRR (40, 41, 43). TRR can (de)catenate DNA efficiently and, together with the helicase BLM, it plays an important role in the dissolution of double Holliday junctions (19, 44–48). In addition, the TRR complex has been proposed to help resolve precatenane structures generated during replication (32).

TRR is also important for resolving ultrafine anaphase bridges (UFBs), which are entangled DNA structures that bridge the separating daughter chromosomes during anaphase (49, 50). The mechanism by which TRR contributes to UFB resolution is thought to depend on the type of UFB that is present (7, 21). For example, UFBs arising from common fragile sites likely contain incompletely replicated DNA, consisting of both double-stranded (ds)DNA and ssDNA (21). It has been proposed that TRR (acting in concert with BLM) plays a direct role in resolving these UFBs due to its abilities to bind to ssDNA and to decatenate DNA (21, 32). In contrast, UFBs linked to centromeres typically consist of fully catenated dsDNA (21). Ultimately, it is believed that the Type 2 topoisomerase TopoIIα is responsible for decatenating centromeric UFBs (21, 51–53). However, there is recent evidence that TRR may also contribute to this pathway, by cooperating with the protein PICH (7, 54), which is an ATP-dependent dsDNA translocase that is found to localize to UFBs (49, 50, 55). Recent studies have shown that the translocation activity of PICH can result in the extrusion of transient dsDNA loops (54, 56). In a torsionally constrained substrate, these loops consist of (hyper)negatively supercoiled DNA, and thus, the DNA adjacent to the loops is positively supercoiled (54). It has been demonstrated that Type 1A topoisomerases (including TRR) can relax the negative supercoils in the loops generated by PICH. This concerted action results in a substrate that is, overall, positively supercoiled. Since positively supercoiled DNA is the preferred substrate for TopoIIα (57, 58), it has been hypothesized that TRR-mediated relaxation of the negatively supercoiled loops generated by PICH may play an important role in the resolution of centromeric UFBs (7, 54).

A recent study using magnetic tweezers has demonstrated that the catalytic cycle of human TopoIIIα exhibits long pause times, during which the enzyme–ssDNA complex is thought to exist in an open, cleaved state (59). Further, it was shown that RMI1 enhances the rate of TopoIIIα binding to ssDNA and acts to stabilize the open, cleaved complex, resulting in longer pause times (59). Nonetheless, the mechanisms by which TRR interacts with and processes negatively supercoiled DNA are still not well understood, especially in the context of its proposed role in UFB resolution.

Here, we report a single-molecule assay to probe the interaction of topoisomerases with negatively supercoiled DNA, which we apply to study the mechanism of supercoil relaxation by human TRR. Our assay exploits a recently developed approach called Optical DNA Supercoiling (ODS), which enables rapid generation of negatively supercoiled DNA by using dual-trap optical tweezers (17, 60). ODS offers several advantages for the study of topoisomerase–DNA interactions. First, the negatively supercoiled substrate can be moved freely between different channels of a microfluidic flow cell, allowing rapid exchange of buffer/protein solutions. Second, ODS is readily compatible with fluorescence microscopy, offering the potential to directly visualize the binding of topoisomerases to the supercoiled DNA substrate. By applying our ODS-based assay, we demonstrate that TRR can relax hypernegatively supercoiled DNA in a highly processive manner, with single complexes capable of performing up to several thousand strand-passage reactions. Our study also indicates that the timescale for TRR-induced relaxation of hypernegatively supercoiled DNA is shorter than the previously reported average lifetime of negatively supercoiled loops generated by PICH (54). These findings provide a rationale for how TRR may relax negatively supercoiled DNA in the context of UFBs.

## Results

### ODS Can Be Used to Measure Real-Time Changes in Supercoiling Density.

**Fig. 1.**
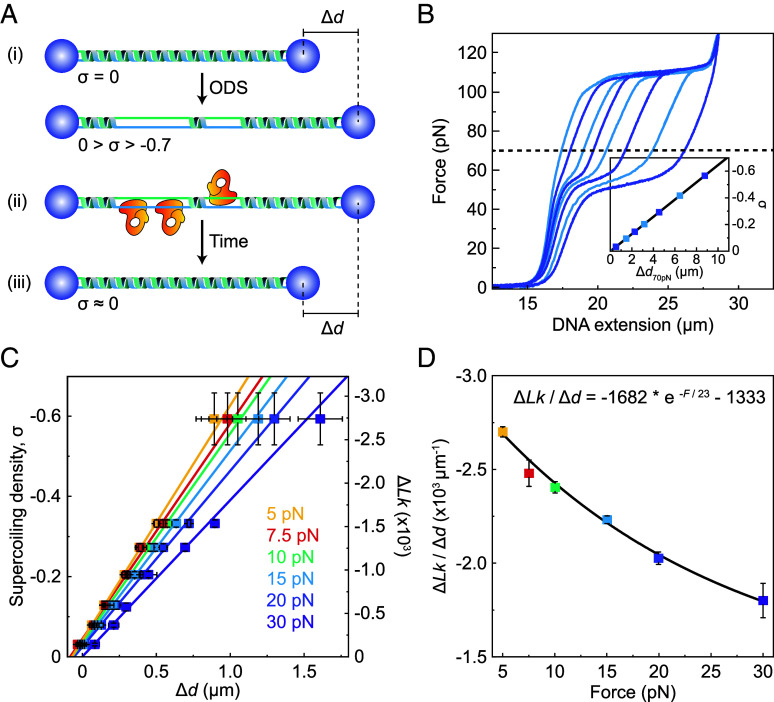
Method to measure supercoil relaxation at different forces using dual-trap optical tweezers. (*A*) Schematic representation of the experimental assay to measure relaxation of a single negatively supercoiled DNA molecule at constant force (≥5 pN). (i) A negatively supercoiled DNA molecule (σ between 0 and ~−0.7), tethered between two optically trapped beads, is generated using ODS. At a constant force above ~5 pN, the length of negatively supercoiled DNA is typically greater than that of B-DNA. This difference in length is referred to here as Δ*d*. (ii) Upon incubating the supercoiled DNA molecule in a solution of Type 1A topoisomerase enzyme (orange), the enzyme can bind to any base-pair melted region that has sufficient ssDNA. (iii) Supercoil relaxation due to topoisomerase activity can be detected in real time by monitoring the decrease in DNA extension (Δ*d*) at a constant force. (*B*) Representative force-extension curves for negatively supercoiled λ-DNA (with different σ) generated via ODS. The black dashed line indicates the change in DNA extension at 70 pN (Δ*d*_70pN_). (*Inset*) Plot showing the change in σ as a function of Δ*d*_70pN_, as determined by comparison with published force-extension curves of negatively supercoiled DNA (17, 60, 61). (*C*) Plot showing the correlation between σ and the change in λ-DNA extension relative to the extension at σ = 0 (Δ*d*) for different forces (ranging between 5 pN and 30 pN). The corresponding change in *Lk* relative to *Lk* at σ = 0 for λ-DNA (Δ*Lk*) is also shown. *N* = 42, grouped into seven mean values of σ. (*D*) Plot showing the ratio of Δ*Lk* to Δ*d* for λ-DNA at forces in the range of 5 pN to 30 pN (corresponding to the gradients of the linear fits in panel *C*). An exponential function is fit to the data, yielding the following empirical relation: Δ*Lk* ≈ (−1,682 * e^−^*^F^*^/23^ − 1,333) * Δ*d.* Error bars in panels *C* and *D* correspond to the ±SE of the mean and fit, respectively.

Our first goal was to establish the experimental framework to measure supercoil relaxation using an ODS-based assay. In these experiments, a single, torsionally constrained λ-DNA molecule was tethered between two optically trapped beads, after which it was negatively supercoiled using ODS. In this assay, a wide range of forces (0 to >150 pN) and supercoiling densities, σ (0 to −0.7) can be applied. Note that σ represents the change in linking number (Δ*Lk*) relative to the linking number of nonsupercoiled DNA (*Lk*_0_), i.e., σ = Δ*Lk*/*Lk*_0_. Next, we exploited the fact that, at a constant force, the DNA extension typically changes as a function of σ (61, 62). Therefore, the relaxation of supercoils is expected to yield a change in the DNA extension (Fig. 1*A*). With reference to published force-distance (FD) curves (61), we have previously shown that the DNA extension at 70 pN (Δ*d*_70pN_) increases as a function of negative supercoiling in an approximately linear manner, for σ between 0 and −0.7 (Fig. 1*B*) (17). In principle, this provides a means to measure real-time changes in σ at 70 pN due to supercoil relaxation. However, topoisomerases are typically force dependent (31, 35, 63) and are expected to exhibit negligible catalytic activity at such a high force. Therefore, to utilize our assay to monitor topoisomerase-mediated supercoil relaxation, it is essential to measure changes in σ at forces much lower than 70 pN. However, the correlation between σ and DNA extension at forces between 5 pN and 70 pN has not been fully characterized. To overcome this, we used a series of FD curves of negatively supercoiled DNA (generated via ODS) where the value of σ was determined based on the extension at 70 pN. Using these FD curves, we then measured the DNA extension at 5 pN, 7.5 pN, 10 pN, 15 pN, 20 pN, and 30 pN. For each force, we observed that the difference in extension between supercoiled and nonsupercoiled DNA (Δ*d*) increases as a function of σ (and thus Δ*Lk*) in an approximately linear manner (Fig. 1*C*). Moreover, the ratio of Δ*Lk* to Δ*d* (determined from the linear fits in Fig. 1*C*) was found to decrease as a function of force in a manner that can be described well by a monoexponential function (Fig. 1*D*). Based on this exponential function, we can determine a value for Δ*Lk*/Δ*d* for any force ≥5 pN. In this way, changes in *Lk* (and thus σ) (e.g., due to topoisomerase activity) can be monitored in real time based on i) the measured DNA extension and ii) the value of Δ*Lk*/Δ*d* at the relevant force.

The ability of our assay to measure changes in σ in real time depends on both the applied force and the initial value of σ. The minimum change in σ that we can detect ranges from 0.007 at 5 pN to 0.002 at 30 pN. This is achievable provided the absolute magnitude of σ is above a critical value (σ_c_), where σ_c_ ranges from ~−0.04 at 5 pN to ~−0.002 at ≥30 pN (*SI Appendix*, Fig. S1). Therefore, to measure very small supercoiling densities (<<−0.04), the applied force should (at least transiently) be fixed at 30 pN (*SI Appendix, SI Note* 1).

### ODS Enables Real-Time Detection of Topoisomerase-Mediated Supercoil Relaxation.

**Fig. 2.**
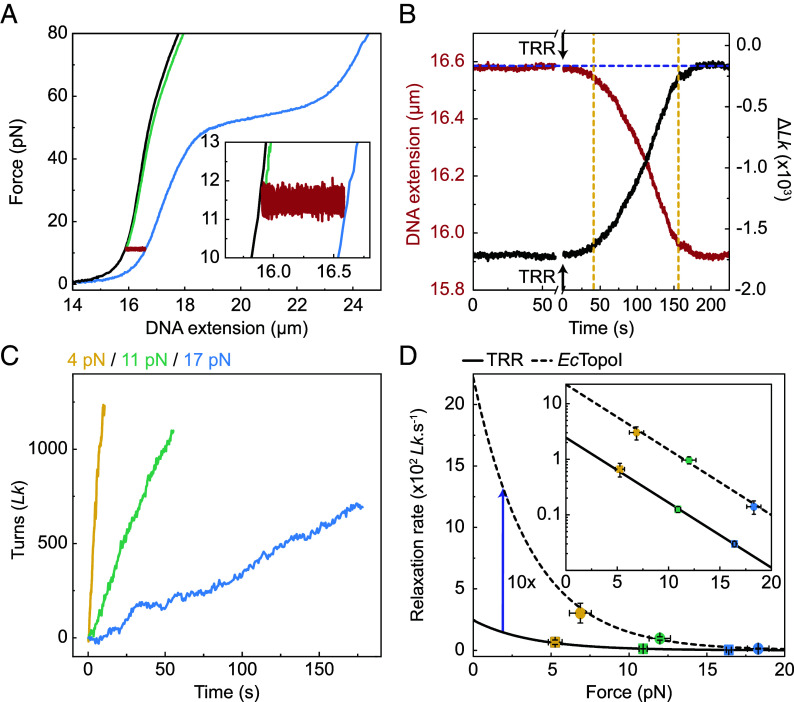
TRR and *Ec*TopoI exhibit different rates of supercoil relaxation, but a similar dependence on force. (*A*) Representative force-extension curves of λ-DNA before (black) and after (blue) the generation of negative supercoiling (σ ~−0.4) by ODS. The red trace shows the decrease in DNA extension upon incubating the supercoiled molecule in a channel containing 10 nM TRR (at 11.5 pN). The force-extension curve recorded after 3 min of incubation in the TRR channel is shown in green. The *Inset* shows an expanded view of the decrease in DNA extension during the 3-min incubation in the TRR channel. (*B*) Change in extension (red curve) for the λ-DNA molecule shown in panel *A* upon incubation in 10 nM TRR (at 11.5 pN). The corresponding change in *Lk* relative to that of nonsupercoiled λ-DNA (Δ*Lk*) is indicated by the black curve. The time at which the DNA molecule was moved to the TRR channel is highlighted by the black arrows. The purple dashed line indicates the minimum supercoiling density that can be detected at this force (~−165 Δ*Lk*) (*SI Appendix*, Fig. S1). The slope of the curves between the yellow dashed lines is approximated as linear (*SI Appendix, SI Methods*). (*C*) Representative traces showing the change in *Lk* over time (associated with the linear Δ*Lk*-time regime, as defined in panel *B*) for negatively supercoiled DNA (with an average initial σ of ~−0.3) in the presence of 10 nM TRR at three different forces. (*D*) Plot showing the rate of supercoil relaxation in the linear Δ*Lk*-time regime (for an average initial σ of ~−0.3) as a function of force in the presence of either 10 nM TRR (solid line) or 10 nM *Ec*TopoI (dashed line). Data were obtained from the slope of the linear region of Δ*Lk*-time plots (e.g., panel *C*). The solid and dashed lines represent fits of the Arrhenius equation to the data. The approximately 10-fold difference in the rate of supercoil relaxation between TRR and *Ec*TopoI is illustrated by the purple arrow. The *Inset* shows the data on a semi-ln scale. Data were obtained from 21 DNA molecules for TRR and from 13 molecules for *Ec*TopoI. Error bars correspond to ±SEM.

We next applied the above calibration approach to measure the rate of supercoil relaxation by human TRR. These experiments were conducted in a multichannel microfluidic flow cell, in which the supercoiled DNA molecule could be moved rapidly between a protein-free channel (containing only measurement buffer) and a channel containing the topoisomerase. We first generated a negatively supercoiled molecule (σ ~−0.4) in the protein-free channel and recorded an FD curve in the same channel (Fig. 2*A*, blue trace). We then incubated the supercoiled molecule in a channel containing 10 nM TRR at a constant force of 11.5 pN for ~250 s. During this time, a substantial reduction in the DNA extension was detected (Fig. 2*A*, red trace), consistent with supercoil relaxation. We then converted the change in DNA extension to the corresponding change in *Lk* (relative to that of nonsupercoiled DNA) (Fig. 2*B*) using the Δ*Lk*/Δ*d* calibration factor at 11.5 pN (determined from Fig. 1*D*). Since bound TRR has negligible influence on the FD curve at a given σ, the presence of the protein does not significantly alter the calibration factor used (*SI Appendix*, Fig. S2 and *SI Note* 2).

The data in Fig. 2*B* reveal that supercoil relaxation proceeded via three distinct regimes: 1) During the first ~40 s (after moving the supercoiled substrate into the TRR channel), the relaxation rate increased progressively, which we attribute to the sequential binding of multiple TRR complexes to the DNA molecule that each contribute to supercoil relaxation (*SI Appendix, SI Note* 3). 2) From ~40 to ~155 s, the relaxation rate was approximately constant, suggesting that the number of bound TRR complexes had reached a steady state. 3) From ~155 s onward, the relaxation rate decreased progressively, and by ~200 s the estimated value of σ was at, or below, our detection limit (σ_c_) at 11.5 pN (i.e., σ between ~−0.04 and 0). We subsequently stretched the DNA molecule to forces >30 pN (Fig. 2*A*, green curve), where our detection limit of σ is −0.002. This revealed that the value of σ after 250 s was −0.009. These results indicate that the supercoil-relaxation rate decreases upon approaching low values of σ, consistent with previous studies of bacterial Type 1A topoisomerases (63–65).

### TRR Exhibits a 10-Fold Lower Rate of Supercoil Relaxation than *E. coli* TopoI.

We next sought to determine how the rate of supercoil relaxation is influenced by the level of applied tension. To this end, we repeated the above experiments at ~5 pN and ~16 pN and calculated the average maximum relaxation rate (based on the linear change in *Lk* as a function of time; see Fig. 2*C* and *SI Appendix, SI Methods*). We then plotted this rate (*v*) as a function of force, *F* (Fig. 2*D*). These data are described well by the Arrhenius law: *v* ∝ e^−^*^F^*^δ/^*^kBT^* (35), where *δ* is the distance to the transition state (and defines the strength of the dependence of *v* on force), and *k_B_T* is the thermal energy. By fitting this function to the data in Fig. 2*D*, we determined that *δ* is 1.1 ± 0.1 nm. We also repeated the above experiments using *E. coli* (*Ec*) TopoI to provide a comparison with TRR using the same substrate (*SI Appendix*, Fig. S3). This revealed that, under our experimental conditions, TRR exhibited an approximately 10-fold lower rate of supercoil relaxation than *Ec*TopoI (Fig. 2*D*). However, both enzymes exhibited a similar dependence on the applied force (with a *δ* value of 1.1 ± 0.1 nm).

**Fig. 3.**
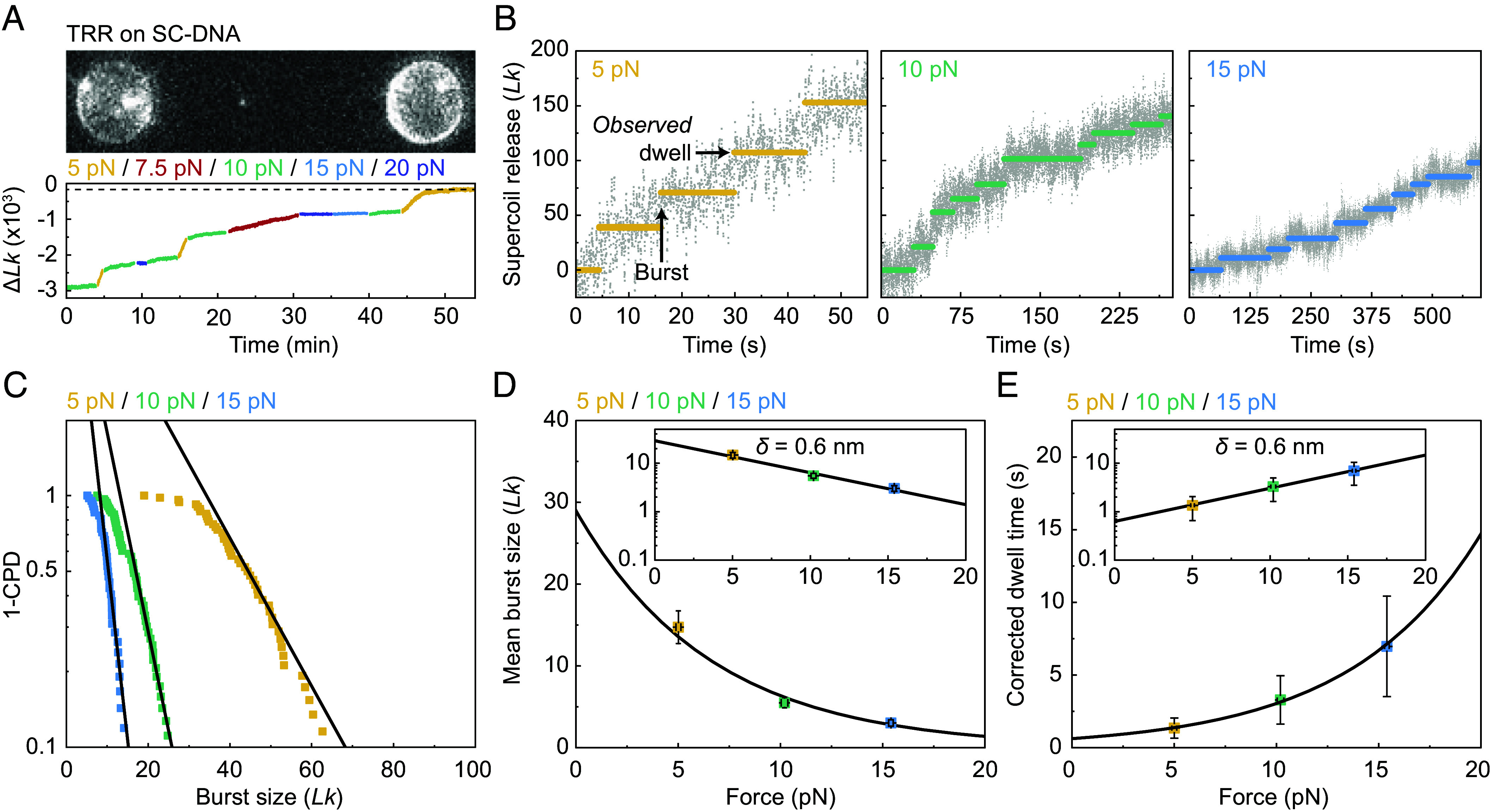
Single TRR complexes can undergo thousands of catalytic cycles via bursts of activity. (*A*, *Top*) A representative fluorescence image showing a single TRR^mCherry^ complex bound to a negatively supercoiled (SC) λ-DNA molecule (with initial σ ~−0.65) tethered between two optically trapped beads. (*Bottom*) Plot showing the corresponding change in *Lk* relative to that of nonsupercoiled λ-DNA (Δ*Lk*) over time due to the action of the single TRR complex shown in the *Top* panel. Note that the applied force was varied during the measurement, as highlighted by the different colors. The dashed line indicates the supercoiling detection limit (~−190 Δ*Lk*) at ~5 pN (*SI Appendix*, Fig. S1). (*B*) Representative traces showing the stepwise change in *Lk* during supercoil relaxation (for σ at least −0.1) by a single TRR complex over time at 5, 10, and 15 pN, respectively. The horizontal lines correspond to the segments detected by an automated segmentation algorithm (*SI Appendix, SI Methods*). The black arrows indicate examples of a burst and an *observed* dwell, respectively. (*C*) 1- Cumulative probability distributions (1-CPD) of the measured burst sizes of TRR at 5, 10, and 15 pN, respectively (semi-ln scale). The black lines represent fits to the fraction of data that can be reliably described by an exponential function (*SI Appendix*, Fig. S6 and *SI Methods*). *N* = 53, 64, and 43 (from seven different DNA molecules) for the data at 5, 10, and 15 pN, respectively. (*D*) Plot showing the mean burst size of TRR as a function of force, as determined from the exponential fits shown in panel *C* (*SI Appendix, SI Methods*). The black line depicts a fit of an exponential function, with the same exponent as the Arrhenius equation, to the data, from which the characteristic distance to the transition state (δ) was extracted. The *Inset* shows the data on a semi-ln scale. Error bars correspond to ±SEM. (*E*) Plot showing the corrected mean dwell time of TRR as a function of force (*SI Appendix, SI Methods*). The black line depicts a fit of an exponential function, with the same exponent as the Arrhenius equation, to the data, from which δ was extracted. The *Inset* shows the data on a semi-ln scale. Error bars correspond to ±SEM.

In the above experiments, we measured the force-dependent rate of supercoil relaxation for multiple TRR complexes acting simultaneously on the supercoiled DNA molecule. We next sought to compare this to the case of a *single* TRR complex. To achieve this, we incubated a negatively supercoiled DNA molecule in a channel containing a low (1 nM) concentration of fluorescently labeled TRR (TRR^mCherry^) for 5 s and then moved the DNA molecule to a protein-free channel. By recording a fluorescence image immediately after moving the substrate into the protein-free channel, we could select for cases where only a single TRR^mCherry^ complex was bound to the supercoiled DNA molecule (based on the fluorescence intensity of a single mCherry fluorophore; see *SI Appendix, SI Methods*). Fig. 3*A* shows a representative fluorescence image of a single TRR^mCherry^ complex bound to a negatively supercoiled DNA molecule, alongside the corresponding change in *Lk* over time under different applied forces (calculated from the change in DNA extension). This figure highlights three important features. First, that a single TRR complex can catalyze thousands of strand passage reactions (at least 3,000 *Lk*) without dissociating from the DNA. Second, the relaxation rate (at a given force) is broadly constant over a wide range of σ (from ~−0.6 to at least ~−0.2, see *SI Appendix*, Fig. S4), but decreases when σ is between −0.1 and 0. Third, as expected based on our “ensemble” TRR experiments in Fig. 2, the rate of supercoil relaxation by a single TRR^mCherry^ complex is force-dependent. To quantify the latter, we plotted the average relaxation rate of single TRR^mCherry^ complexes as a function of force and fitted the Arrhenius equation to the data (*SI Appendix*, Fig. S5). The value of *δ* determined from this fit (1.4 ± 0.1 nm) was similar to that obtained from ensemble measurements. Nonetheless, the overall rate of relaxation in ensemble experiments was ~20-fold higher than for a single TRR complex. This, in turn, suggests that there were approximately 20 TRR complexes acting on the DNA molecule in the ensemble experiments in Fig. 2.

### TRR Relaxes Negatively Supercoiled DNA with High Processivity.

An advantage of measuring supercoil relaxation by single TRR complexes is that the processivity of the enzyme can be determined. As highlighted in Fig. 3*B*, the relaxation of negatively supercoiled DNA by a single TRR complex proceeds via bursts of processive activity (consisting of multiple consecutive changes in *Lk*), separated by periods of apparent inactivity (dwell times). This is similar to the behavior reported for several other Type 1A topoisomerases, based on magnetic tweezers studies (9, 35, 37, 66, 67). These previous studies revealed that the burst sizes and dwell times can vary markedly between different Type 1A homologs. A recent magnetic tweezers study of human TopoIIIα on a DNA substrate with low supercoiling density (0 < σ < −0.06) at 0.8 pN and containing a 20 bp mismatch reported that the burst size is ~1.5 *Lk*, while the duration of the first pause is ~15 s (59). The same study found that the duration of the first pause increases to ~20 s in the presence of RMI1. However, the processivity of eukaryotic Type 1A topoisomerases is still much less characterized, including at higher supercoiling densities and at different forces. To quantify the processivity of TRR, we segmented the change in *Lk* over time (induced by a single TRR complex) into a series of bursts and dwells (*SI Appendix, SI Methods*). Dwell times were ascribed to durations where *Lk* remained constant (within our distance resolution; see below for more details), while the size of each burst was determined from the difference in *Lk* between successive dwells (Fig. 3*B*).

Due to the processive nature of Type 1A topoisomerases (9, 26, 35, 37, 66, 68), the distribution of burst sizes is expected to be exponential. This is because the catalytic cycle proceeds in steps of 1 *Lk* and the size of a burst depends on the number of times the enzyme can capture the T-strand and perform strand transfer without pausing. However, an exponential distribution is only observed above a critical burst size, as highlighted by the linear region on a semi-ln plot (Fig. 3*C*). This is due to the distance resolution of our instrument, in which the minimum burst size that can be resolved ranges from ~20 *Lk* at 5 pN to ~10 *Lk* at 15 pN. We therefore identified the subset of data that could be described reliably by an exponential function. This was defined as the data for which the fit had a coefficient of determination (*R*^2^) > 0.98 (69) and a fractional error <2 times the minimum fractional error (*SI Appendix*, Fig. S6 and *SI Methods*). Based on these fits, we determined the mean burst size to be 14.7 ± 2.0 *Lk*, 5.5 ± 0.6 *Lk* and 3.0 ± 0.4 *Lk* at 5 pN, 10 pN, and 15 pN, respectively. Furthermore, by fitting a plot of burst size as a function of force (Fig. 3*D*) to an exponential function with the same exponent as the Arrhenius equation (i.e., *F*δ/*k_B_T*), we found that the force dependence of the burst size is roughly half the force dependence of the overall relaxation rate (*δ* = 0.6 ± 0.1 nm versus 1.1 ± 0.1 nm). While our assay cannot resolve individual strand passage events, our results are consistent with TRR using an enzyme-bridged strand passage mechanism for supercoil relaxation (*SI Appendix, SI Note* 4).

Since we cannot resolve bursts below a critical threshold, our *observed* dwell times include unresolved bursts and are therefore longer than the *corrected* dwell times. To correct for this, we first estimated the average number of unresolved bursts per observed dwell (*N*_unresolved_*/dwell*) at each force. This was achieved by extrapolating the exponential fits in Fig. 3*C* to a burst size of 1 *Lk* and calculating the number of bursts required to recapitulate this function (*SI Appendix, SI Methods*). The corrected dwell time was then estimated by subtracting *N*_unresolved_*/dwell* + 1 from the observed dwell time. This yielded *corrected* mean dwell times of 1 ± 1, 3 ± 2, and 7 ± 3 s at 5 pN, 10 pN, and 15 pN, respectively (Fig. 3*E*). By fitting a plot of *corrected* dwell time as a function of force to an exponential function with the same exponent as the Arrhenius equation (*F*δ/*k_B_T*), we obtained a *δ* value of 0.6 ± 0.1 nm, approximately half that associated with the overall relaxation rate and equal to the *δ* value obtained for the burst size. This indicates that the burst size and dwell time are equally sensitive to force.

To evaluate the robustness of the above analysis, we calculated the average rate of supercoil relaxation associated with a single TRR complex at a given force by dividing the mean burst size by the corrected dwell time. We then compared this with the relaxation rate determined by dividing the *total* change in *Lk* by the duration of the measurement (*SI Appendix*, Fig. S7). Both approaches yielded similar relaxation rates, indicating that our assay (based on the combination of ODS and fluorescence imaging) provides a robust approach to determine the mean burst size and dwell time for single TRR complexes.

### Direct Observation of Sequence-Dependent Binding to Negatively Supercoiled DNA.

**Fig. 4.**
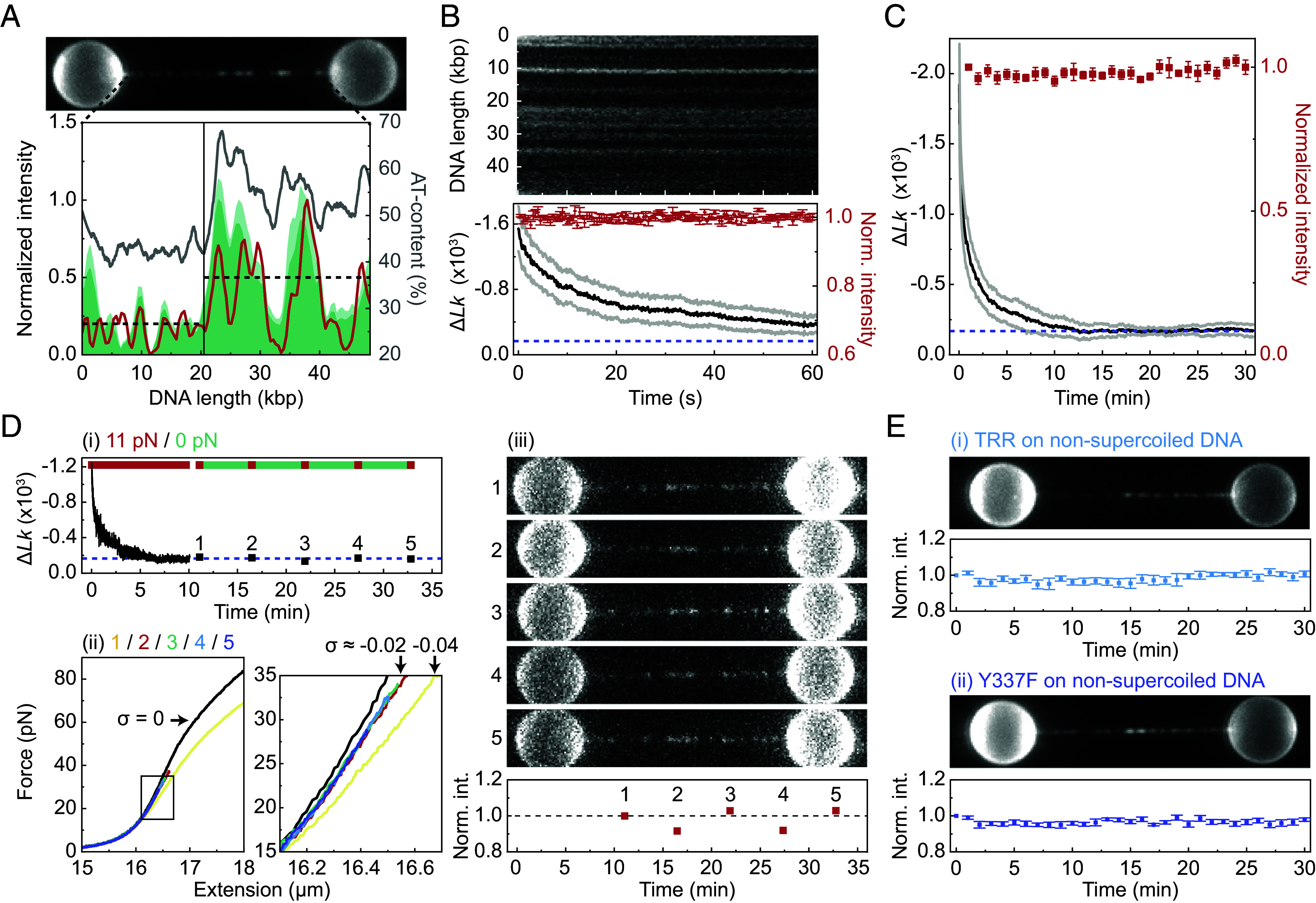
TRR remains bound to negatively supercoiled DNA after supercoil relaxation. (*A*, *Top*) Representative fluorescence image showing multiple (~50) TRR^mCherry^ complexes bound to a negatively supercoiled λ-DNA molecule (σ ~−0.4) at 10 pN. (*Bottom*) Plot showing the normalized fluorescence intensity profile of TRR^mCherry^ bound to negatively supercoiled λ-DNA (σ ~−0.4). The red trace corresponds to the fluorescence image in the *Top* panel. The dark and light green indicate the mean and mean+SEM fluorescence intensity profile, respectively, for nine negatively supercoiled λ-DNA molecules. The average AT-content of λ-DNA (GenBank: J02459) is shown in gray. The vertical black line marks the division of λ-DNA into regions with low and high AT-content, while the horizontal dashed lines show the average TRR^mCherry^ intensity in each region. (*B*, *Top*) Representative kymograph showing the TRR^mCherry^ fluorescence signal along a negatively supercoiled λ-DNA molecule (initial σ ~−0.4) over a period of 60 s at ~10 pN. The kymograph was recorded in a protein-free channel, following incubation of the supercoiled molecule in 10 nM TRR^mCherry^ for 30 s. (*Bottom*) Plot showing the change in *Lk* relative to that of nonsupercoiled λ-DNA (Δ*Lk*) for negatively supercoiled λ-DNA (initial σ ~−0.4) in a protein-free channel at ~10 pN, following incubation in 10 nM TRR^mCherry^ for 30 s. The mean Δ*Lk* and ±SEM (for five molecules) are shown in black and gray, respectively. The corresponding (bleaching-corrected) normalized mCherry fluorescence intensity is shown in red. The purple dashed line indicates the supercoiling detection limit (~−165 Δ*Lk*) at ~10 pN (*SI Appendix*, Fig. S1). (*C*) Plot showing the mean Δ*Lk* and ±SEM (in black and gray, respectively) for five negatively supercoiled λ-DNA molecules (initial σ ~−0.3) during a 30-min period in a protein-free channel at ~10 pN, following 30 s incubation in 10 nM TRR^mCherry^. The corresponding (bleaching-corrected) normalized mCherry fluorescence intensity is shown in red. The purple dashed line indicates the supercoiling detection limit (~−165 Δ*Lk*) at 10 pN. (*D*, *i*) Representative trace showing Δ*Lk* over time for a negatively supercoiled λ-DNA molecule (initial σ ~−0.3) in a protein-free channel following 30 s incubation in 10 nM TRR^mCherry^. After ~10 min at 11 pN in the protein-free channel (time-point 1), the force was reduced to 0 pN for a duration of ~20 min. During this 20-min period, a force-extension curve and a fluorescence image (the latter at ~10 pN) were recorded once every ~5 min (at time-points 2-5). The purple dashed line indicates the detection limit (~−165 Δ*Lk*) at ~10 pN. (*ii*, *Left*) Force-extension curves corresponding to the DNA molecule in (i), recorded at time-points 1-5. (*Right*) Expanded view of the black box in the *Left* panel. (*iii*, *Top*) Fluorescence images of TRR^mCherry^ bound to the DNA molecule in (i) and (ii) at time points 1-5. (*Bottom*) Plot showing the bleaching-corrected normalized mCherry fluorescence intensity for the images shown in the *Top* panel. The experiments reported in panel D were repeated three times, with similar results on each occasion. (*E*) Fluorescence signal for (i) TRR^mCherry^ and (ii) TRR^Y337F-mCherry^ bound to a nonsupercoiled torsionally constrained λ-DNA molecule in a protein-free channel at 10 pN, following incubation of the DNA molecule in 10 nM protein for 30 s at ~120 pN. For each case, a representative mCherry fluorescence image is shown, alongside the bleaching-corrected normalized fluorescence intensity over time, averaged over 5 molecules. Error bars correspond to ±SEM.

Since Type 1A topoisomerases require ssDNA for catalytic activity (10–12), it is anticipated that TRR will bind to regions of negatively supercoiled DNA that show the greatest tendency for strand separation. Although base-pair melting occurs preferentially in AT-rich sequences (13, 14, 16, 17), underwound DNA can potentially adopt a range of topological conformations (15, 62, 70–72), to which TRR may have different abilities to bind. It is therefore important to determine how TRR binding depends on the local sequence composition. To test this, we first incubated a negatively supercoiled DNA molecule (σ ~−0.4) at 10 pN for 30 s in a channel containing 10 nM TRR^mCherry^. The supercoiled molecule was then moved to a protein-free channel, where an mCherry fluorescence image was recorded. In each case, TRR^mCherry^ was present primarily on one half of the supercoiled DNA (as highlighted in Fig. 4*A*). Note that TRR has negligible affinity for dsDNA (21), and thus the binding is associated with regions of ssDNA. Since the direction of the DNA molecule in our setup is random, we oriented each fluorescence image such that the asymmetric fluorescence intensity profiles were coaligned. We then calculated the average fluorescence intensity profile across all aligned supercoiled molecules (dark green trace, Fig. 4*A*). By comparing this intensity trace to the profile of the average AT-content along the λ-DNA molecule (gray trace, Fig. 4*A*), we found that the fluorescence intensity (and thus the number of TRR complexes) is ~threefold higher in the AT-rich half of the DNA molecule than the GC-rich half. We also observed that TRR exhibits a similar sequence-dependent binding profile on overstretched nonsupercoiled λ-DNA (*SI Appendix*, Fig. S8). These sequence-dependent binding profiles are similar to those reported previously for the ssDNA binding protein RPA, which is known to bind base-pair melted bubbles (17). Taken together, our assay allowed us to directly confirm the hypothesis that TRR binds preferentially to supercoiling-induced bubbles at AT-rich sequences.

### TRR Remains Bound to DNA Long after Supercoils Are Relaxed.

Given that a single TRR complex can perform many relaxation bursts without dissociating from the DNA (Fig. 3*A*), we sought to establish if, and to what extent, supercoil relaxation induces dissociation of TRR from the DNA. To this end, we first incubated a negatively supercoiled substrate (σ in the range of −0.3 to −0.4) in a channel containing 10 nM TRR^mCherry^ for 30 s at 30 pN to allow the binding of multiple TRR^mCherry^ complexes. We then moved the supercoiled molecule to a protein-free channel and quantified the change in TRR^mCherry^ fluorescence intensity over time at 10 pN, while simultaneously measuring the change in *Lk* due to supercoil relaxation. Fig. 4*B* shows an example where we imaged the TRR^mCherry^ fluorescence continuously over a period of 1 min. During this time, σ reduced by >75%, yet the fluorescence intensity remained constant (after correcting for photobleaching, see *SI Appendix, SI Methods*). In other cases, we monitored the TRR^mCherry^ fluorescence signal over much longer periods of time (up to 30 min) by recording a fluorescence snapshot every minute. An example of this is shown in Fig. 4*C*. In this example, most of the supercoiling density was relaxed within the first 2 min. After ~15 to 20 min, the relaxation rate was negligible and the calculated value of σ was below our detection limit at 10 pN. Strikingly, the TRR^mCherry^ fluorescence signal remained constant over the full 30-min experiment, indicating that, in a protein-free buffer channel, most TRR complexes remained bound to the DNA for >10 min after the point when σ reached our detection limit.

Similar to the data obtained in 10 nM unlabeled TRR (Fig. 2), the relaxation rate in Fig. 4 *B*–*D* decreases substantially at low σ. However, the value of σ at which the relaxation rate starts to decrease in Fig. 4 *B*–*D* is notably higher than in Fig. 2*B*, and it takes longer for Δ*Lk* to reach the detection limit in Fig. 4 *B*–*D*. We anticipate that the TRR coverage is higher in Fig. 4 *B*–*D* than in Fig. 2, owing to the fact that the DNA was incubated in the TRR channel for 30 s at high force (~30 pN). Under these conditions, substantial protein binding can occur with minimal supercoil relaxation (relaxation in Fig. 4 *B*–*D* is permitted once the DNA molecule has been moved to a protein-free channel and the force reduced to ~10 pN). In contrast, in Fig. 2*B*, the force was ~10 pN at all times (and relaxation was monitored inside the protein channel); as a result, it is likely that some relaxation (and thus loss of bubbles) will have occurred in Fig. 2*B* before the TRR coverage could reach saturation. This is in agreement with our conclusion that ~50 TRR complexes were bound to the DNA in Fig. 4 *A*–*D* (based on fluorescence intensities), whereas ~20 TRR complexes were bound to the DNA in Fig. 2*B* (based on a comparison with the relaxation rates for a single TRR). Taken together, this suggests that the precise manner in which the relaxation rate decreases at low σ depends on the number of bound TRR (*SI Appendix, SI Note* 3).

We next examined whether reducing the tension could promote TRR to dissociate from the DNA. To this end, we first incubated a negatively supercoiled DNA molecule in the TRR channel (10 nM) and then moved the supercoiled substrate to a protein-free channel at ~10 pN. After ~10 min at this force, the rate of supercoil relaxation had reduced to near zero (Fig. 4 *D*, *i*). Next, we held the DNA at 0 pN for 20 min, interrupting this only briefly to stretch the molecule every ~5 min to measure Δ*Lk* (with high precision, at ~30 pN) and to record a fluorescence snapshot (Fig. 4*D*). This experiment yielded two important observations. First, ~5 min after reducing the force from ~10 pN to 0 pN, the remaining supercoiling density had decreased slightly, from σ = −0.04 to σ = −0.02 but then stayed at this value for the next ~15 min (Fig. 4 *D*, *ii*). Second, during the entire experiment, dissociation of TRR from the DNA was not detected (i.e., the TRR^mCherry^ fluorescence signal was constant in each snapshot (Fig. 4 *D*, *iii*). To confirm that at least some of the bound TRR complexes were still catalytically active, we generated more negative supercoiling (by performing ODS in the buffer channel) and detected supercoil relaxation by the bound TRR (*SI Appendix*, Fig. S9).

Given that TRR has negligible binding affinity to dsDNA (21), we hypothesized that the lack of TRR dissociation may indicate that TRR stabilizes partially denatured structures in the DNA. To test this, we incubated an overstretched, nonsupercoiled, torsionally constrained DNA molecule (which contains base-pair melted “bubble” structures) in a channel containing 10 nM TRR^mCherry^ for 30 s at ~120 pN, and then moved the molecule to a protein-free channel before decreasing the tension to 10 pN. We observed that TRR did not dissociate upon decreasing the tension to 10 pN in the protein-free buffer channel, even after 30 min (Fig. 4 *E*, *i*). This indicates that TRR stabilizes bubble structures within the DNA, independent of whether the molecule is supercoiled or not. We also repeated the above experiments, but using a catalytically inactive mutant of TRR (26) (TRR^Y337F-mCherry^), which is able to bind to, but not cleave, ssDNA (21, 32). We found that this mutant also remained bound to nonsupercoiled DNA for at least 30 min after reducing the tension from 120 pN to 10 pN (Fig. 4 *E*, *ii*). This indicates that TRR-induced stabilization of bubble structures in the DNA does not depend on the ability of the enzyme to cleave the DNA backbone.

## Discussion

Here, we have established a single-molecule strategy to study the interaction of Type 1A topoisomerases with negatively supercoiled DNA in real time using a combination of dual-trap optical tweezers and fluorescence imaging. A key feature of our assay is the ability to move the supercoiled molecule between different solutions (using a multichannel flow cell) and to image the binding of topoisomerase along the length of the DNA molecule, while concurrently measuring the change in *Lk*. This has enabled us to provide unique insight into how TRR interacts with and relaxes negatively supercoiled DNA.

The recent discovery that TRR can relax negative supercoils within DNA loops extruded by PICH (resulting in an accumulation of net positive supercoils) has raised the possibility that this pathway may contribute to UFB resolution through stimulating decatenation by TopoIIα (54). Using magnetic tweezers, Bizard et al. demonstrated that, at 2 pN, the rate of loop extrusion by PICH is 82 ± 31 bp s^−1^, with a corresponding processivity of 107 ± 25 bp (54). As a result, the average lifetime of a loop is only ~1.3 s. It has also been demonstrated that the value of σ associated with each loop is −0.6 ± 0.3 (54), resulting in an average Δ*Lk* of ~6.1. It follows, therefore, that the average rate of supercoil relaxation would have to be ~4.7 *Lk* s^−1^ in order to relax all supercoils during the lifetime of the loop. It is important to note that, even if there is tension applied to the DNA substrate, the extruded loop is not expected to be under tension. Our study indicates that, in the absence of tension, the relaxation rate of a single TRR is ~35 *Lk* s^−1^ (at least for σ values >0.1) (*SI Appendix*, Fig. S5), which suggests that a single TRR can relax the negative supercoils within each loop before the loop is released by PICH. This, in turn, supports the hypothesis that the concerted action between PICH and TRR represents an efficient means to induce positive supercoiling during the dissolution of centromeric UFBs. We note that the relaxation rate determined for a single TRR complex in our study assumes that there are no unlabeled proteins (lacking the mCherry-RMI2 subunit) bound to the DNA. Based on previous studies of TRR (32), coupled with the SDS-PAGE of the TRR^mCherry^ sample (*SI Appendix*, Fig. S10), we estimate that the vast majority of the TRR sample (at least 80%) contains all three subunits (*SI Appendix, SI Note* 5).

When considering the processivity of TRR, useful mechanistic insights can be derived from comparing its burst size and dwell time to those reported previously for other Type 1A topoisomerases. When using a negatively supercoiled substrate at 0.7 pN, Terekhova et al. reported that for *Ec*TopoI and *Ec*TopoIII the mean burst size is 20 ± 1.9 *Lk* and 28 ± 4 *Lk*, respectively, while the mean dwell time is 5 s and 114 s, respectively (37). Our results (Fig. 3) indicate that the burst size of TRR at the same force is broadly similar (~30 *Lk*), while the dwell time is notably shorter (~1 s). The termination of a burst is hypothesized to correspond to the enzyme losing its grip on the T-strand, and thus, the dwell time likely represents the time before the enzyme can recapture the T-strand (9, 30). The observed changes in burst size and dwell time as a function of tension are thought to be due to the steric constraint imposed by the tension, which is likely to hinder the ability of the enzyme to maintain its grip on, and recapture, the T-strand. Since the overall rate of relaxation for TRR is ~10-fold lower than for *Ec*TopoI, the shorter dwell time of TRR in our study compared with that reported previously for *Ec*TopoI and *Ec*TopoIII is likely due to the difference in the substrates used between the two studies. In our case, supercoil relaxation was measured at σ values between -0.1 and -0.4, whereas Terekhova et al. measured relaxation over the range 0 < σ < −0.04 (37). Our substrates are expected to contain more flexible underwound DNA structures than the substrates used by Terekhova et al. (*SI Appendix, SI Note* 6), which likely enables the T-strand to be more easily captured. We anticipate that the supercoiled substrates used in our study more closely resemble the topology associated with supercoiled loops within UFBs (as discussed above). Nonetheless, it is important to appreciate that supercoil relaxation at very low σ (between −0.002 and −0.04) can still be detected in our assay by transiently increasing the force to 30 pN and measuring the DNA length at that force (e.g., Fig. 4*D*).

The ability of our assay to move the supercoiled molecule between different buffer solutions and directly image single proteins bound to the DNA allowed us to demonstrate that single TRR complexes can perform several thousand catalytic cycles (>3,000) without dissociating from the DNA. This supports previous single-molecule studies that have indicated that *Ec*TopoI remains bound during dwells (30). Our data also reveal that in a protein-free solution, most DNA-bound TRR complexes remain bound for >30 min after the majority of the supercoiling has been relaxed (at which point the relaxation rate has decreased to near zero). It remains to be determined whether this behavior (which is independent of catalytic activity) is common to other Type 1A topoisomerases. However, in support of this, we note that *Tm*TopoI can actively stabilize underwound DNA at the expense of plectonemes (63). Nonetheless, the long residence time of bound TRR may not be a fundamental feature of the mechanism in the context of the cellular environment. We anticipate that dissociation of TRR from DNA after supercoil relaxation may be promoted by other proteins, as persistent TRR binding could be deleterious for genome stability. One possibility is that TRR dissociation could be induced through a “facilitated dissociation” process via other proteins that compete for DNA binding or even free TRR proteins in solution (73). It has also been reported that positive supercoiling can displace *Tm*TopoI from DNA (63) and, therefore, the concerted action of TRR and PICH (which together generate positive supercoiling) might act to promote dissociation of other TRR complexes from the DNA. Furthermore, TRR is known to form a complex with BLM (40, 74, 75), and the presence of BLM has been shown to alter the mechanical properties of the TRR-ssDNA open gate (32). It is therefore conceivable that BLM could also play a role in promoting dissociation of TRR. It will be important for future studies to unravel the relative contributions that each of these processes may play in removing TRR after supercoil relaxation.

The ability of our assay to visualize the interactions of Type 1A topoisomerases with underwound DNA and concomitantly measure supercoil relaxation in real time provides a powerful platform for future studies to explore how partner proteins regulate the activity of these enzymes. Moreover, although the current work is focused on Type 1A topoisomerases, we envision that the combination of ODS, microfluidics, and fluorescence imaging could also be widely applied to study other families of topoisomerases.

## Materials and Methods

### Sample Preparation.

All reagents were purchased from Sigma-Aldrich unless stated otherwise. End-closed λ-DNA (48,502 bp) was prepared by ligating biotinylated end-caps (Biolegio) to each terminal *cos*-site of λ-DNA (17, 60). Human TRR complexes (TRR^unlabeled^, TRR^mCherry^, and TRR^Y337F-mCherry^) were prepared as described previously (21). In brief, RMI1, RMI2, and wild-type or catalytically dead (Y337F) TopoIIIα were overexpressed in *E. coli* Rosetta 2 (DE3, pLysS, Novagen) cells and purified as a preformed complex. The coding sequence of mCherry was cloned on the N terminus of RMI2. Purified TRR complexes were snap frozen in liquid nitrogen and stored at −80 °C in aliquots (10 µL of 1 µM) in 50 mM Tris-HCl (pH 7.5), 200 mM NaCl, 1 mM DTT, and 20% glycerol. *E. coli* TopoI (M0301L, 920 nM) was purchased from New England Biolabs. In the Y337F mutant, the catalytic tyrosine is replaced by a phenylalanine, and this substitution has been shown to abrogate the catalytic activity required for strand passage and supercoil relaxation in both TRR and other topoisomerases (21, 26, 32, 63).

### Experimental Setup.

Experiments were conducted in a 5-channel microfluidic flow cell positioned on an automated xy-stage within a custom-built inverted microscope that combines dual-trap optical tweezers and wide-field fluorescence microscopy (17, 60, 76). Channels 1 and 2 of the flow cell contained streptavidin-coated polystyrene beads (diameter 4.5 μm, Spherotech) and biotin-labeled end-closed λ-DNA, respectively. Channel 5 contained either TRR or *Ec*TopoI. All other channels contained buffer only. Channels 1 and 2 were used to tether a single λ-DNA molecule between two optically trapped beads (77), and the tethered DNA molecule could be exchanged rapidly between the other channels. The DNA extension was determined using bright-field imaging of the optically trapped beads, while the force applied to the DNA (via displacement of a tethered bead) was measured using back-focal plane interferometry of the condenser top lens using a position-sensitive detector. For constant force experiments, a force-feedback loop was used. The optical tweezers and microfluidics hardware were controlled with a custom-written program written in LabVIEW. For fluorescence imaging experiments, a 561 nm excitation laser source was used (Cobalt Jive 25 mW CW) and the excitation power was controlled using an acousto-optical tunable filter (AA Opto Electronic). Fluorescence was imaged on an EMCCD camera chip (iXON+ 987E, Andor Technology) with a pixel size of 130 nm, using a gain of 50. The acousto-optical tunable filter and EMCCD camera were controlled using Micro-Manager (78).

### Experimental Conditions.

All single-molecule data were obtained at room temperature (~20 °C). Unless stated otherwise, all channels of the microfluidic flow cell contained “measurement buffer”, which consisted of 20 mM Tris-HCl (pH 7.5), 50 mM NaCl, 0.3 mM MgCl_2_, and 0.005% (v/v) Tween-20. The protein channel (channel 5) additionally contained 10 nM TRR^unlabeled^, 10 nM *Ec*TopoI^unlabeled^, 1 nM TRR^mCherry^, 10 nM TRR^mCherry^, or 10 nM TRR^Y337F-mCherry^, depending on the experiment (specified in the figure captions). Channel 5 also contained 1 mM DTT. We note that increasing the magnesium concentration in the measurement buffer to 2 mM did not affect the supercoil-relaxation rate of TRR substantially (*SI Appendix*, Fig. S11). To determine the rate of supercoil relaxation, we measured the change in DNA extension at a constant force, using a force clamp. A reliable force clamp in dual-trap optical tweezers is only possible at forces above a few pN; however, the relaxation rate (as well as burst size and dwell time) at forces less than a few pN can still be estimated by extrapolating the fits to the data obtained between 5 pN and 20 pN (e.g., in Figs. 2*D* and 3*D* and *SI Appendix,* Fig. S5). The uncertainty in determining both the *absolute* value of σ generated by ODS and the relative change in σ (due to relaxation) is described in the *SI Appendix, SI Note* 1.

## Supplementary Material

Appendix 01 (PDF)

Dataset S01 (XLSX)

Dataset S02 (XLSX)

Dataset S03 (XLSX)

Dataset S04 (XLSX)

Dataset S05 (XLSX)

Dataset S06 (XLSX)

Dataset S07 (XLSX)

Dataset S08 (XLSX)

Dataset S09 (XLSX)

Dataset S10 (XLSX)

Dataset S11 (XLSX)

Dataset S12 (XLSX)

Dataset S13 (XLSX)

Dataset S14 (XLSX)

Dataset S15 (XLSX)

## Data Availability

Study data are included in the article and/or supporting information.
