# Supplementary material for: Mechanistic basis for relaxation of DNA supercoils by human topoisomerase IIIα–RMI1–RMI2
Source: Proc Natl Acad Sci U S A. 2026 Jan 23;123(4):e2406949123. doi: 10.1073/pnas.2406949123 (PMC12849704; doi:10.1073/pnas.2406949123)
Supplement: Supplementary file 1 — Appendix 01 (PDF) [file pnas.2406949123.sapp.pdf]

Supporting Information for

Mechanistic basis for relaxation of DNA supercoils by human Topoisomerase IIIα-RMI1-RMI2

Dian Spakman[a], Andreas S. Biebricher[a], Anna H. Bizard[b], Ian D. Hickson[b], Erwin J.G. Peterman[a,1,2], Gijs J.L. Wuite[a,1,2], Graeme A. King[c,1,2]

[a] *Department of Physics and Astronomy, and LaserLaB Amsterdam, Vrije Universiteit Amsterdam, De Boelelaan 1081, 1081 HV, Amsterdam, The Netherlands.*

[b] *Center for Chromosome Stability, Department of Cellular and Molecular Medicine, University of Copenhagen, Blegdamsvej 3B, 2200 Copenhagen N, Denmark.*

[c] *Institute of Structural and Molecular Biology, Division of Biosciences, University College London, Gower Street, London WC1E 6BT, United Kingdom.*

[1] *To whom correspondence should be addressed. Email: e.j.g.peterman@vu.nl; g.j.l.wuite@vu.nl; g.king@ucl.ac.uk.*

[2] *These authors contributed equally to this work.*

This PDF file includes:

Supporting Information text: SI Methods
Supporting Information text: SI Notes 1-6
Figures S1 to S11
Legends for Datasets S1 to S15
SI References

Other supporting materials for this manuscript include the following:

Datasets S1 to S15

Supporting Information text: SI Methods

Determination of TRR / *Ec*TopoI relaxation rate in ensemble experiments. The rate of supercoil relaxation in the presence of 10 nM $TRR^{unlabeled}$ or $EcTopoI^{unlabeled}$ (Fig. 2 and Fig. S3, respectively) was determined from the slope of the linear region of the change in *Lk* as a function of time. The presence of three distinct regimes involving (i) a gradual increase in relaxation rate, (ii) a linear relaxation rate and (iii) a decrease in relaxation rate at low supercoiling densities was observed frequently at various forces. The start of the linear regime was assumed to correspond to the point at which the number of bound TRR complexes had reached a steady state. We defined the linear regime as the region of the *Lk versus* time plot for which there was a continuous increase in *Lk* greater than 2 times the standard deviation of the signal in the absence of protein. A linear function was then fitted to the linear region of the plot, and the slope of this fit was considered to be the average maximum relaxation rate.

Generation of TRR binding profiles. Fluorescence intensity profiles of $TRR^{mCherry}$ bound to DNA were obtained by measuring the total mCherry fluorescence intensity at each x-axis pixel along a line (with 5-pixel width) that spans the length of the DNA molecule. Each profile was corrected for background fluorescence by subtracting an average of two fluorescence intensity profiles along a line with the same length and width as described above, but positioned above and below the DNA molecule, respectively.

Monitoring the coverage of TRR on DNA over time. A fluorescence snapshot of $TRR^{mCherry}$ (or $TRR^{Y337F\text{-}mCherry}$) bound to DNA was recorded with 1 second exposure either every second (Fig. 4B), every minute (Fig. 4C and 4E) or approximately every 5 minutes (Fig. 4D). The total fluorescence intensity in each frame (I_0) was calculated by summing the intensity of the background-corrected fluorescence intensity profile along the DNA molecule. The contribution of photobleaching to the measured fluorescence intensity was subtracted using the following equation: $I(N_{\text{frame}}) = I_0 + (1 - e^{N_{\text{frame}}/\tau})$, where I is the mCherry fluorescence intensity in the absence of photobleaching, τ is the photobleaching rate (described below) and N_{frame} is the frame number. If dissociation of TRR from DNA occurs, this will correspond to a change in I. When plotting the fluorescence intensity in Fig. 4B, 4C and 4E, all data were normalized to the fluorescence intensity in the first frame.

Measuring the mCherry photobleaching rate. A molecule of overstretched λ-DNA was incubated in a channel that contained 10 nM $TRR^{mCherry}$ and then moved to a protein-free buffer channel where the mCherry fluorescence was imaged. Since TRR does not dissociate from ssDNA (and thus overstretched DNA) over a period of at least tens of minutes,[1] the decrease in fluorescence intensity in this case is due solely to photobleaching. The photobleaching rate was determined from an exponential fit to a plot of the total bound $TRR^{mCherry}$ fluorescence intensity as function of frame number.

Identifying single mCherry fluorophores. Single $TRR^{mCherry}$ complexes bound to the DNA were identified based on the magnitude of a single photobleaching step, following published approaches.[2–4]

Determination of the TRR burst size. A segmentation algorithm (written in MATLAB, R2015b, The MathWorks Inc,[5] and available in Bakx *et al.*[1]) was used to identify discrete steps in the distance-time traces due to a single $TRR^{mCherry}$ complex. The threshold for a discrete burst was set as four times the standard deviation of the baseline signal (determined in the absence of protein). The burst durations were similar to our time resolution (<0.2 seconds) and therefore the segmentation algorithm assumes that each burst occurs instantaneously. Note that, for this reason, it is not possible to determine burst reaction rates from our data. The ΔLk associated with each measured burst was calculated using the $\Delta Lk/\Delta d$ values determined from Fig. 1D. We then plotted the 1- Cumulative Probability Distribution (1-CPD) of the measured burst sizes for each force (Fig. S6A). Next, we identified the sub-set of this distribution that could be described reliably by an exponential function. To this end, we progressively reduced the maximum burst size included in the fit, to generate a series of data sets, each with an accompanying fit to an exponential function. We defined a reliable fit as having a coefficient of determination (R^2) >0.98

(Fig. S6B) and where the fractional error associated with (a) the exponential decay parameter and (b) the extrapolated value of 1-CPD at 1 *Lk* was <2 times the minimum fractional error (Fig. S6C). For each data set that met the fitting criteria, we extracted the exponential decay parameter, resulting in a set of exponential decay parameters for each force (Fig. S6C). These decay parameters were then averaged, which yielded a measure of the average burst size at each force. Similarly, for each data set that met the fitting criteria, we also extracted the value of 1-CPD at 1 *Lk* (corresponding to a single catalytic cycle [6]) (Fig. S6C). These values were averaged to yield a measure of the average 1-CPD at 1 *Lk* for each force. We then calculated the total number of unresolved bursts ($N_{unresolved}$), i.e., the number of bursts that would be required to recapitulate each exponential function to *Lk* = 1. Following this, we divided $N_{unresolved}$ by the total measurement time, to determine the number of unresolved bursts per dwell ($N_{unresolved}/dwell$). The corrected dwell time was then determined by dividing the mean *observed* dwell time (determined from the segmentation algorithm) by $N_{unresolved}/dwell + 1$. The uncertainty associated with the corrected dwell time was estimated by propagating the standard error of the mean *observed* dwell time and the standard error of the mean value of $N_{unresolved}/dwell$.

Statistical analysis. All data analyses were performed using OriginPro 2019, unless specified otherwise. All errors associated with fitting parameters represent the standard error of the fit. All other errors correspond to the standard error of the mean (SEM). Fluorescence images were processed using ImageJ based on published codes.[7]

Supporting Information text: SI Notes

SI Note 1: Determination of the minimum change in σ that can be detected using our assay.

The uncertainty in determining the *absolute* value of σ generated by ODS was estimated to be ~0.03 to 0.045.[8] The precision with which we can measure a *relative* change in σ is fundamentally limited by the distance resolution of our instrument, which is ~10 nm at 5 pN and ~5 nm at 30 pN. Therefore, based on the magnitude of $\Delta Lk/\Delta d$, the minimum relative change in σ that we can detect ranges from 0.007 at 5 pN to 0.002 at 30 pN. However, this is only valid above a critical value of σ (σ_c), which ranges from ~-0.04 at 5 pN to ~0 at ≥30 pN (Fig. S1). This is because there is no detectable difference in the DNA extension between σ=0 and σ_c, and thus the linear relationship between σ and Δd determined in Fig. 1C only applies above σ_c. For this reason, small supercoiling densities (between -0.04 and -0.002) can only be detected in our assay at forces ≥30 pN.

SI Note 2: Binding of TRR to underwound DNA has negligible influence on DNA length at a given σ.

We have demonstrated previously that the binding of multiple TRR complexes to λ-ssDNA can lead to substantial (up to 50%) lengthening of the molecule, due to the formation of multiple open TRR-ssDNA gates, which each increase the DNA length ~8.5 nm.[1] In the current study, we estimate that up to ~50 TRR complexes were bound to underwound regions of both negatively supercoiled DNA and overstretched dsDNA. If each of these TRR complexes were to increase the DNA length by 8.5 nm, the DNA molecule would be ~0.4 μm longer than expected at a given σ. Since our measurement of σ is based on the length of the DNA molecule, any perturbation to the DNA length at a given σ due to TRR binding could result in an incorrect determination of σ.

To establish if TRR alters the length of underwound DNA (independent of supercoil relaxation), we incubated non-supercoiled, torsionally constrained, λ-dsDNA in 10 nM TRR at ~120 pN. At this force (where the DNA is overstretched), TRR binds extensively to underwound regions present in the molecule (Fig. 4E). We then reduced the force to ~10 pN and recorded a FD-curve. As shown in Fig. 4E, TRR remains bound to the DNA when the force is reduced from 120 pN to 10 pN. Fig. S2 compares the average FD-curve of non-supercoiled λ-dsDNA following incubation in 10 nM TRR at 120 pN with the average FD-curve of non-supercoiled λ-dsDNA in the absence of TRR. This revealed that TRR induces a small length increase of the DNA molecule (~0.015 μm at 30 pN). Using the equation in Fig. 1D, this change in length corresponds to an 'apparent' σ of ~-0.006 (even though the molecule is non-supercoiled). The magnitude in the discrepancy of σ is only slightly greater than the smallest change in σ that can be detected in our assay at 30 pN (~-0.002). Thus, any length increase of underwound DNA due to the binding of TRR is sufficiently small that it has negligible impact on our determination of σ.

The above observation indicates that TRR induces substantially less lengthening of underwound DNA than of a native ssDNA molecule. We therefore conclude that the presence of a second strand in underwound DNA limits the extent to which the molecule can be lengthened.

SI Note 3: It is unlikely that two TRR complexes can bind to opposite strands of a single bubble in underwound DNA.

Since TRR requires only ssDNA for binding, it is anticipated that binding can occur on either strand of an underwound DNA molecule. Nonetheless, it is unlikely that two TRR complexes can bind *directly* opposite to each other in a *single* bubble for the following reasons:

(i) Once one TRR complex is bound to one strand of a bubble, it would likely be difficult for a second TRR complex to bind directly opposite to this (i.e., on the other stand of the bubble) due to steric constraint.

(ii) If two TRR complexes were bound to opposite strands of a single bubble, we might expect this to lead to a much more elongated substrate (due to TRR-induced gate opening of the ssDNA on *each* strand of the bubble). However, while extensive lengthening occurs on ssDNA, it does not occur on underwound DNA (Fig. S2 and SI Note 2).

SI Note 4: Relaxation of supercoils by TRR is expected to occur via an enzyme-bridged strand passage mechanism under our experimental conditions.

There is compelling evidence that the relaxation of supercoils by Type 1A topoisomerases occurs via an enzyme-bridged strand passage mechanism, in which the enzyme holds onto both ends of the cleaved strand, creating a gate through which the non-cleaved strand can pass.[1,6,9] This mechanism necessitates that relaxation should occur in steps of 1 *Lk*. In contrast, Type 1B topoisomerases relax supercoils via a rotation mechanism. In this case, the enzyme holds onto only one end of the cleaved strand, allowing the other end to rotate freely around the intact DNA strand, and thus supercoil relaxation occurs in steps of *n Lk*.[10]

In our experiments, we observe that the relaxation of supercoils by TRR proceeds via a series of bursts (composed of multiple consecutive relaxation steps) separated by pauses. This behavior has also been reported for several other Type 1A topoisomerases.[6,11–14] Due to the spatial and temporal resolution of our assay, we cannot resolve individual relaxation steps and thus we cannot rule out the possibility that TRR relaxes supercoils via a rotation mechanism under our experimental conditions. Nonetheless, we consider this to be unlikely for the following reasons:

1) We have demonstrated previously that TRR forms an enzyme-mediated gate in ssDNA and the enzyme maintains its grip on both ends of the cleaved strand until forces >30 pN,[1] well above the forces used in our relaxation experiments reported here.

2) If free rotation of the cleaved strand were to occur during supercoil relaxation, it would render the DNA molecule torsionally unconstrained (at least transiently). Such behaviour would be detectable from the FD-curves: torsionally unconstrained DNA exhibits a sharp transition into overstretching and the overstretching plateau occurs at 65 pN. This was never observed in our experiments; rather, the FD-curves are consistent with the DNA being torsionally constrained at all times (see, for example, Fig. S9).

SI Note 5: The TRR complex is stable under our experimental conditions.

To scan for DNA molecules that contained a single bound TRR complex (e.g., Fig. 3), we selected cases where only a single mCherry fluorophore was present (note that the mCherry label is on the RMI2 subunit). We cannot completely exclude the possibility that a fraction of our protein sample lacks the (labeled) RMI2 subunit, but any such fraction is expected to be very small for the following reasons:

1) When incubating λ-ssDNA in a high concentration of $TRR^{mCherry}$, the DNA is extensively coated with protein (as determined from mCherry fluorescence images).[1] Moreover, this is correlated with a substantial length increase of the ssDNA (reflecting the opening of multiple topoisomerase-mediated gates in the ssDNA).[1] These observations indicate that (i) the vast majority of $RMI2^{mCherry}$ is bound to the topoisomerase enzyme and (ii) little, if any, unlabeled TopoIIIα or TopoIIIα-RMI1 is present on the DNA.

2) When probing the interaction of $BLM^{SNAP649}$ with $TRR^{mCherry}$ on ssDNA, a strong colocalization between SNAP649 and mCherry fluorescence was observed.[1] Given that BLM can interact with both TopoIIIα and TRR,[15] this supports the conclusion that little, if any, unlabeled TopoIIIα or TopoIIIα-RMI1 is present on the DNA.

3) Based on the Coomassie staining in the SDS PAGE shown in Fig. S10 (taking into account the molecular weight of the three subunits), the stoichiometry of our $TRR^{mCherry}$ sample was calculated to be 1.0:0.8:0.8. Given the uncertainty in quantifying the stoichiometry based on

Coomassie staining, coupled with points 1 and 2 above, we conclude that the vast majority of the sample (at least 80%) contains all three subunits.

SI Note 6: Comparison of the force dependence of TRR and *Ec*TopoI with previous studies.

Our experiments demonstrate that both TRR and *Ec*TopoI exhibit a similar dependence on force, with a force-dependency factor (δ) of 1.1±0.1 nm. Previous studies of *Ec*TopoI and *Thermotoga maritima* (*Tm*)TopoI have reported that δ is ~10 nm when using a positively supercoiled substrate containing a 12 nucleotide (nt) mismatch, but that there is negligible force-dependence when the mismatch is replaced by a 25 nt bulge of ssDNA.[6,16] On the basis of these observations, it was proposed that tension introduces a steric constraint that hinders the conformational changes necessary for strand passage, and the presence of a bulge reduces this constraint due to its inherent flexibility.[6,16] Given that the value of δ for TRR and *Ec*TopoI in our study is an order of magnitude lower than that reported previously for *Ec*TopoI on a 12 nt mismatch, we postulate that the underwound structures present in our substrates have a high flexibility, similar to that associated with a 25 nt bulge.

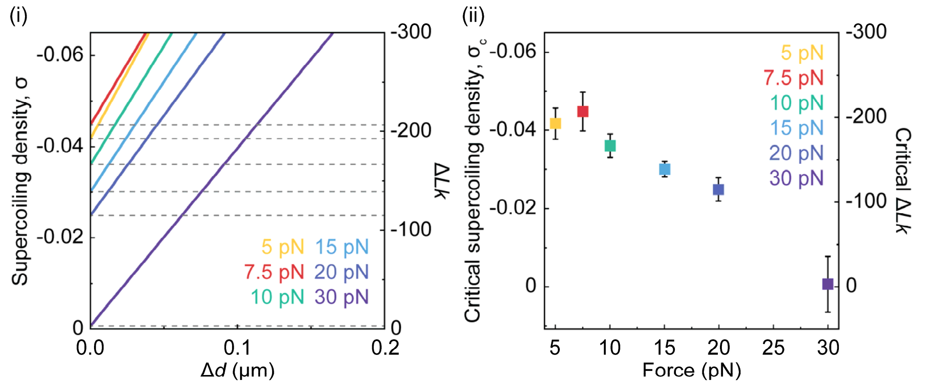

Fig. S1. Y-axis intercepts for the linear fits in Fig. 1C show a dependence on force. (i) Reproduction of the linear fits in Fig. 1C, showing the correlation between σ (and thus Δ*Lk*) and Δ*d* (the change in DNA extension relative to that of non-supercoiled λ-DNA) for forces between 5 pN and 30 pN. The dashed lines highlight the y-axis intercept for each fit and represent the critical supercoiling density, σ_c (i.e., the minimum supercoiling density that we can detect based on the difference in length between supercoiled and non-supercoiled DNA) at each force. (ii) Plot showing σ_c (the y-axis intercepts in panel (i)) and the corresponding Δ*Lk* as a function of force. Error bars represent the ±SE of the fits.

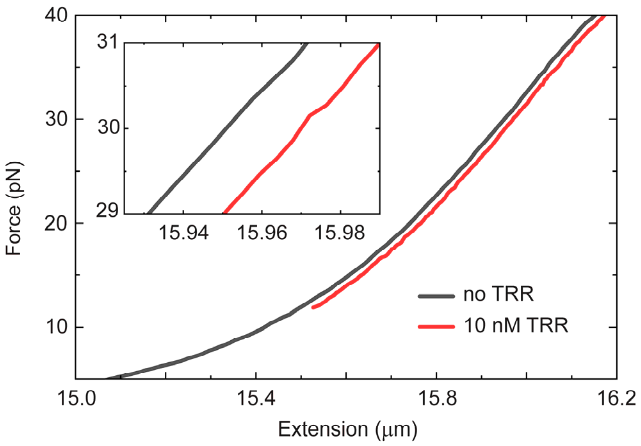

Fig. S2. The presence of TRR bound to underwound DNA has a negligible impact on our ability to measure σ. Plot shows the average FD-curve for non-supercoiled, torsionally constrained λ-DNA in the absence of TRR (black trace) and the average FD-curve for the same substrate following incubation in 10 nM TRR for 30 seconds at ~120 pN (red trace). As demonstrated in Fig. 4E, TRR binds extensively to underwound regions of overstretched non-supercoiled DNA and remains bound even when the force is reduced to 10 pN. Inset highlights the small degree of DNA lengthening at low forces due to bound TRR. At 30 pN, the increase in length is ~0.015 μm which, using the equation in Fig. 1D, corresponds to an 'apparent' σ of ~-0.006, even though the molecule is non-supercoiled. This difference between the 'real' and 'apparent' value of σ is close to the minimum difference in σ that can be detected using our assay at 30 pN (~-0.002, see SI Note 1). We therefore conclude that TRR-induced lengthening of underwound DNA is sufficiently small that it has negligible impact on our ability to reliably measure σ. See SI Note 2 for more details. Note that each average FD-curve is derived from 6 individual FD-curves recorded under identical stretching conditions.

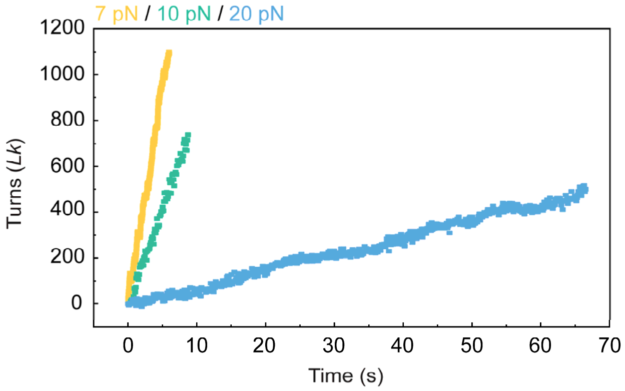

Fig. S3. Sample traces showing that supercoil relaxation by *Ec*TopoI is force dependent. Representative traces showing the change in *Lk* over time for negatively supercoiled λ-DNA (with an initial σ of ~-0.3) in the presence of 10 nM *Ec*TopoI at three different forces. These traces correspond to the linear Δ*Lk*-time regime, as defined in Fig. 2B (see also SI Methods).

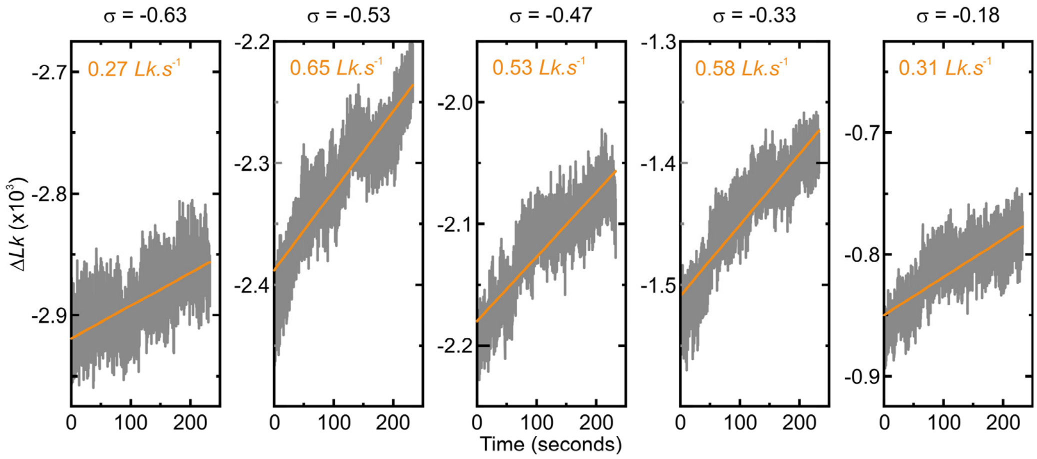

Fig. S4. The average rate of supercoil relaxation for a single TRR complex is broadly constant across a wide range of σ. Plots show the change in *Lk* relative to that of non-supercoiled λ-DNA (Δ*Lk*) over time due to the action of a single TRR complex at 10 pN, derived from the trace shown in Fig. 3A. The value of σ stated above each plot indicates the initial σ at the start of each plot. The orange lines are linear fits to the data. The corresponding relaxation rate determined from each fit is stated in orange. While there is some variation in the rate between each panel, there is no trend as a function of σ.

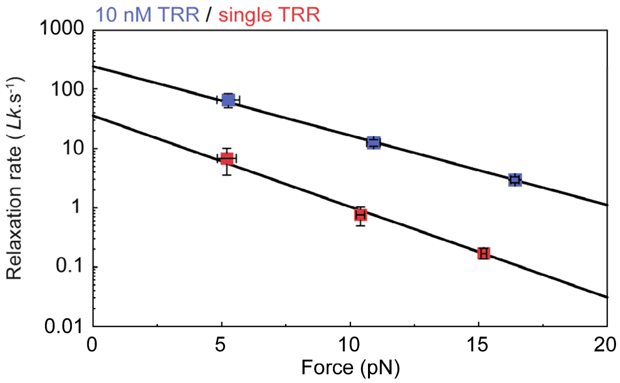

Fig. S5. The dependence of the supercoil-relaxation rate on force is similar for single-TRR and ensemble-TRR experiments. Plot shows the mean rate of supercoil relaxation as a function of force for ensemble experiments (conducted in the presence of 10 nM TRR, purple) and single-TRR experiments (in which a single TRR complex is bound to the supercoiled DNA molecule, red). Black lines represent fits of the Arrhenius equation to the data. N = 21 and N = 15 for ensemble-TRR and single-TRR experiments, respectively. Error bars correspond to ±SEM.

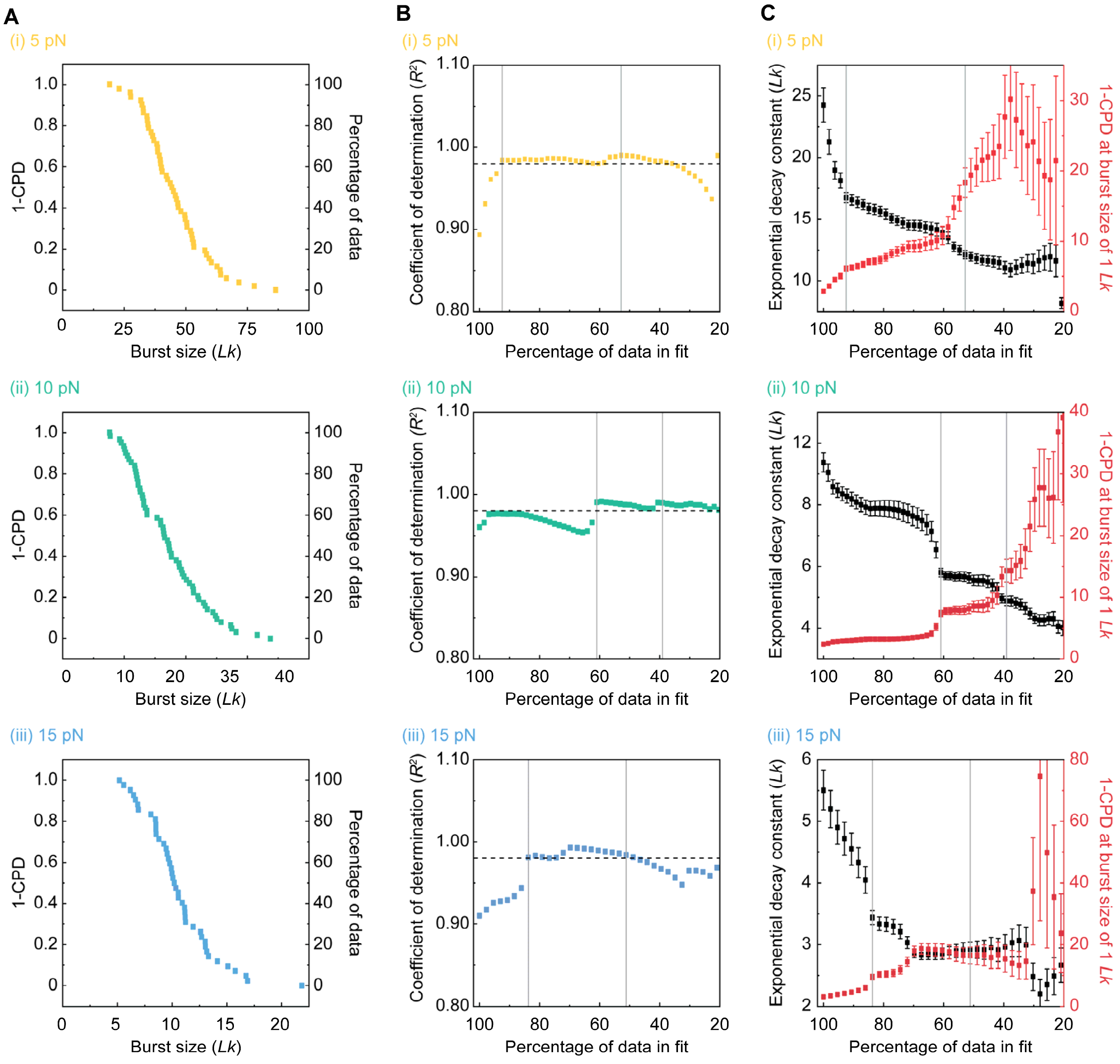

Fig. S6. Analytical approach used to identify the subset of the 1-CPD *vs* burst size data that can be described reliably by an exponential function. (A) Plots showing the 1 - Cumulative probability distribution (1-CPD) of the measured burst sizes, and the corresponding percentage of data above a given burst size, at 5 pN (i), 10 pN (ii) and 15 pN (iii). (B) Plots showing the coefficient of determination (R^2) for exponential fits to the data in panel A, as a function of the fraction of the dataset included in the fit, for 5 pN (i), 10 pN (ii) and 15 pN (iii). The horizontal dashed lines indicate R^2 = 0.98. (C) Plots showing the exponential decay constant (black) and the extrapolated value for 1-CPD at Lk = 1 (red) for exponential fits to the data in panel A, as a function of the fraction of the dataset included in the fit, for 5 pN (i), 10 pN (ii) and 15 pN (iii). Error bars correspond to the absolute errors of the fit (±SE). The vertical solid lines in panels B and C highlight the fraction of data for which both R^2 is >0.98 and the fractional error associated with (a) the exponential decay parameter and (b) 1-CPD at Lk = 1 is <2 times the minimum fractional error. The mean burst size and the mean value of 1-CPD at Lk = 1 was determined using the exponential fits to the data between the two vertical lines in panels B and C (see SI Methods).

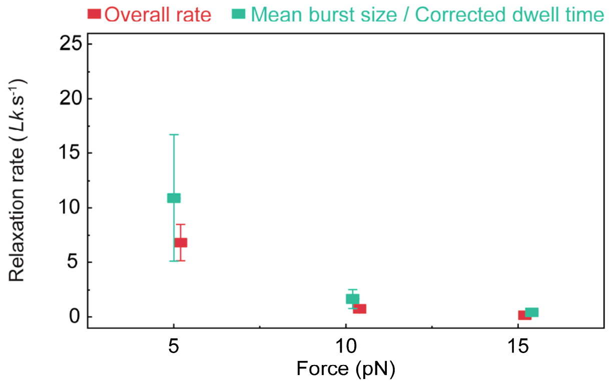

Fig. S7. The overall rate of supercoil relaxation by a single TRR complex is similar to the ratio of the mean burst size to the corrected dwell time for TRR. Plot shows the mean rate of supercoil relaxation for a single TRR complex as a function of force, determined by either measuring the change in *Lk* over time (red) or dividing the mean burst size by the corrected mean dwell time (green). Error bars correspond to ±SEM.

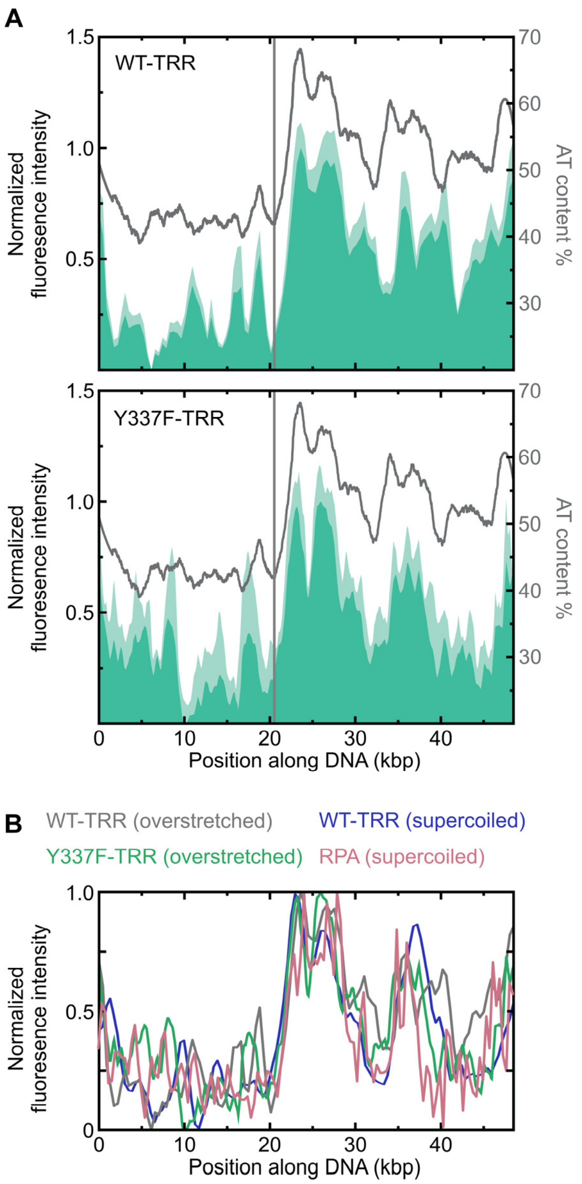

Fig. S8. Binding of TRR to overstretched non-supercoiled DNA occurs primarily in AT-rich sequences. (A) Plots showing the normalized mean fluorescence intensity profile (dark green) of wild-type $TRR^{mCherry}$ (*top*) and $TRR^{Y337F\text{-}mCherry}$ (*bottom*) along non-supercoiled λ-DNA at 10 pN, following incubation of the DNA molecule in 10 nM wild-type $TRR^{mCherry}$ and $TRR^{Y337F\text{-}mCherry}$, respectively, at ~120 pN. The corresponding mean+SEM is shown in light green and the average AT-content of λ-DNA is displayed in grey (GenBank: J02459). The vertical black line marks the division of λ-DNA into regions with low and high AT-content. Data were derived from 5 DNA molecules for wild-type $TRR^{mCherry}$ and 4 DNA molecules for $TRR^{Y337F\text{-}mCherry}$. (B) Plot showing the normalized mean fluorescence intensity profiles for (i) wild-type $TRR^{mCherry}$ along non-supercoiled DNA at 10 pN following incubation of the DNA molecule in 10 nM $TRR^{mCherry}$ at 120 pN (grey), (ii) $TRR^{Y337F\text{-}mCherry}$ along non-supercoiled DNA at 10 pN following incubation of the DNA molecule in 10 nM $TRR^{Y337F\text{-}mCherry}$ at 120 pN (green), (iii) wild-type $TRR^{mCherry}$ along negatively supercoiled DNA (σ ~-0.4) at 10 pN (purple), taken from Fig. 4A and (iv) RPA along negatively supercoiled DNA (σ ~-0.69) at 5 pN (pink), derived from King *et al.*[8]

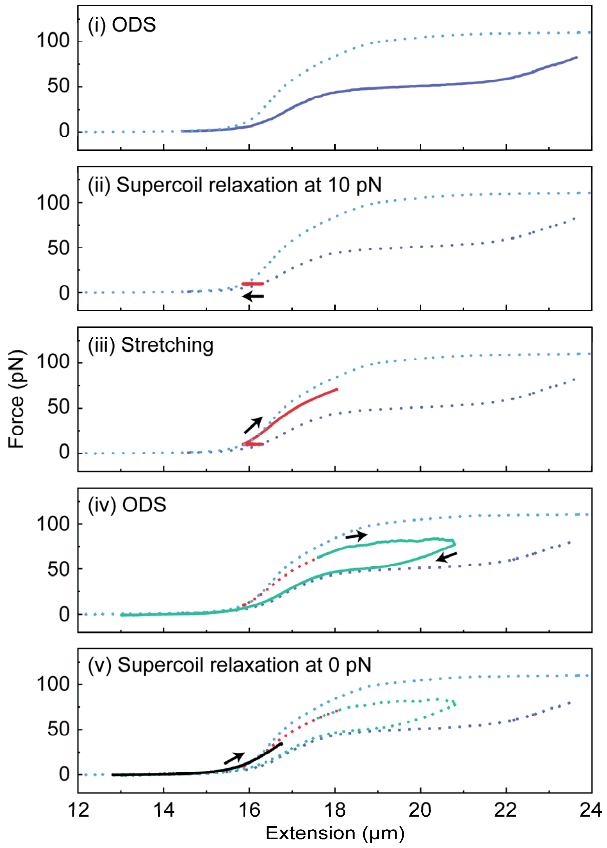

Fig. S9. TRR that remains bound to DNA following supercoil relaxation is still catalytically active. Plots show a series of sequential force-extension curves for λ-DNA recorded before and after incubation in 10 nM TRR. (i) A negatively supercoiled DNA molecule (σ ~-0.3) was generated in a protein-free channel by ODS. The force-extension curves recorded before and after ODS are shown in dotted and solid lines, respectively. (ii) The supercoiled DNA molecule was moved to a channel containing 10 nM TRR and incubated at 10 pN for ~30 seconds. The DNA molecule was then moved back to a protein-free channel. The red trace shows the change in DNA extension (due to supercoil relaxation by bound TRR) at 10 pN over a period of ~10 minutes in the protein-free channel. (iii) After monitoring the change in extension for ~10 minutes, a force-extension curve was recorded (up to ~70 pN, red) in the protein-free channel. This confirmed that much of the supercoiling generated in panel (i) had been relaxed. (iv) With the DNA molecule still in the protein-free channel (but bound by TRR), the supercoiling density was increased again by performing ODS (as indicated by the green force-extension curves). (v) Immediately after performing ODS, the tension was decreased to 0 pN and, after a few seconds at zero force, a force-extension curve was recorded (black curve). This revealed that most of the supercoiling generated in the protein-free channel (in panel (iv)) was relaxed in a few seconds at 0 pN by the TRR complexes bound to the DNA molecule. The black arrows indicate the directions of the force-extension curves.

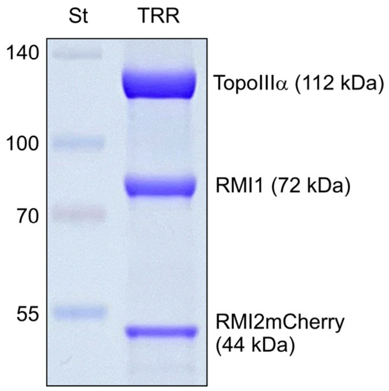

Fig. S10. SDS-PAGE of the recombinant $TRR^{mCherry}$ used in this study. Lane 1 shows a series of molecular weight standards (‘St’), in kDa. Lane 2 shows the $TRR^{mCherry}$ sample, with the three subunits marked. The molecular weights of the $TRR^{mCherry}$ subunits are 112, 72 and 44 kDa, respectively, including the tags. TopoIIIα has 6xhis and thrombin cleavage tags, RMI1 has a 6xhis tag and RMI2 has the mCherry tag. The calculated ratio of the three bands in Lane 2 (based on Coomassie staining, and corrected for molecular weight) is 1.0:0.8:0.8.

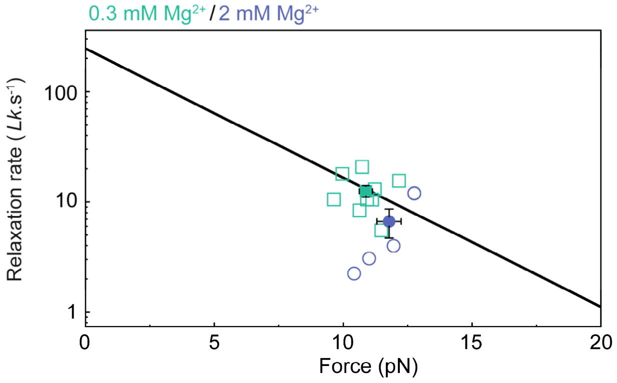

Fig. S11. The rate of supercoil relaxation by TRR does not depend strongly on whether the $MgCl_2$ concentration is 0.3 mM or 2 mM. Plot shows the rate of supercoil relaxation at forces between ~9 pN and ~13 pN in the presence of 10 nM TRR, with either 0.3 mM $MgCl_2$ (green) or 2 mM $MgCl_2$ (purple) included in the measurement buffer. The open symbols represent the rates for individual molecules, whereas the solid symbols correspond to the mean rate for each $MgCl_2$ concentration (averaged over the force range). Data were obtained from 9 DNA molecules for 0.3 mM Mg^{2+} and 5 DNA molecules for 2 mM Mg^{2+}. Error bars correspond to ±SEM. The black line shows the fit of the Arrhenius equation to the data obtained for 0.3 mM Mg^{2+}, reproduced from Fig. 2D.

Dataset S1 (separate file). Data used to compile Fig. 1

Dataset S2 (separate file). Data used to compile Fig. 2

Dataset S3 (separate file). Data used to compile Fig. 3

Dataset S4 (separate file). Data used to compile Fig. 4

Dataset S5 (separate file). Data used to compile Fig. S1

Dataset S6 (separate file). Data used to compile Fig. S2

Dataset S7 (separate file). Data used to compile Fig. S3

Dataset S8 (separate file). Data used to compile Fig. S4

Dataset S9 (separate file). Data used to compile Fig. S5

Dataset S10 (separate file). Data used to compile Fig. S6

Dataset S11 (separate file). Data used to compile Fig. S7

Dataset S12 (separate file). Data used to compile Fig. S8

Dataset S13 (separate file). Data used to compile Fig. S9

Dataset S14 (separate file). Data used to compile Fig. S10

Dataset S15 (separate file). Data used to compile Fig. S11

SI References

1. J. A. M. Bakx, A. S. Biebricher, G. A. King, P. Christodoulis, K. Sarlós, A. H. Bizard, I. D. Hickson, G. J. L. Wuite, E. J. G. Peterman, Duplex DNA and BLM regulate gate opening by the human TopoIIIα-RMI1-RMI2 complex. *Nat. Commun.* 13, 584 (2022).
2. K. Sarlós, A. S. Biebricher, A. H. Bizard, J. A. M. Bakx, A. G. Ferreté-Bonastre, M. Modesti, M. Paramasivam, Q. Yao, E. J. G. Peterman, G. J. L. Wuite, I. D. Hickson, Reconstitution of anaphase DNA bridge recognition and disjunction. *Nat. Struct. Mol. Biol.* 25, 868–876 (2018).
3. A. S. Biebricher, S. Hirano, J. H. Enzlin, N. Wiechens, W. W. Streicher, D. Huttner, L. H.-C. Wang, E. A. Nigg, T. Owen-Hughes, Y. Liu, E. J. G. Peterman, G. J. L. Wuite, I. D. Hickson, PICH: A DNA translocase specially adapted for processing anaphase bridge DNA. *Mol. Cell* 51, 691–701 (2013).
4. J. Van Mameren, M. Modesti, R. Kanaar, C. Wyman, E. J. G. Peterman, G. J. L. Wuite, Counting RAD51 proteins disassembling from nucleoprotein filaments under tension. *Nature* 457, 745–748 (2009).
5. J. W. J. Kerssemakers, E. L. Munteanu, L. Laan, T. L. Noetzel, M. E. Janson, M. Dogterom, Assembly dynamics of microtubules at molecular resolution. *Nature* 442, 709–712 (2006).
6. N. H. Dekker, V. V. Rybenkov, M. Duguet, N. J. Crisona, N. R. Cozzarelli, D. Bensimon, V. Croquette, The mechanism of type IA topoisomerases. *Proc. Natl. Acad. Sci. U.S.A.* 99, 12126–12131 (2002).
7. C. A. Schneider, W. S. Rasband, K. W. Eliceiri, NIH Image to ImageJ: 25 years of image analysis. *Nat. Methods* 9, 671–675 (2012).
8. G. A. King, F. Burla, E. J. G. Peterman, G. J. L. Wuite, Supercoiling DNA optically. *Proc. Natl. Acad. Sci. U.S.A.* 116, 26534–26539 (2019).
9. M. Mills, Y.-C. Tse-Dinh, K. C. Neuman, Direct observation of topoisomerase IA gate dynamics. *Nat. Struct. Mol. Biol.* 25, 1111–1118 (2018).
10. N. M. Baker, R. Rajan, A. Mondraǵon, Structural studies of type I topoisomerases. *Nucleic Acids Res.* 37, 693–701 (2009).
11. D. Spakman, J. A. M. Bakx, A. S. Biebricher, E. J. G. Peterman, G. J. L. Wuite, G. A. King, Unravelling the mechanisms of type 1A topoisomerases using single-molecule approaches. *Nucleic Acids Res.* 49, 5470–5492 (2021).
12. K. Terekhova, K. H. Gunn, J. F. Marko, A. Mondragón, Bacterial topoisomerase I and topoisomerase III relax supercoiled DNA via distinct pathways. *Nucleic Acids Res.* 40, 10432–10440 (2012).
13. A. Strzałka, M. J. Szafran, T. Strick, D. Jakimowicz, C-terminal lysine repeats in Streptomyces topoisomerase I stabilize the enzyme-DNA complex and confer high enzyme processivity. *Nucleic Acids Res.* 45, 11908–11924 (2017).
14. X. Yang, S. Saha, W. Yang, K. C. Neuman, Y. Pommier, Structural and biochemical basis for DNA and RNA catalysis by human topoisomerase 3β. *Nat. Commun.* 13, 4656 (2022).
15. L. Wu, S. L. Davies, P. S. North, H. Goulaouic, J.-F. Riou, H. Turley, K. C. Gatter, I. D. Hickson, The Bloom’s syndrome gene product interacts with topoisomerase III. *J. Biol Chem*. 275, 9636–9644 (2000).
16. N. H. Dekker, T. Viard, C. B. de La Tour, M. Duguet, D. Bensimon, V. Croquette, Thermophilic topoisomerase I on a single DNA molecule. *J. Mol. Biol.* 329, 271–282 (2003).
